# A NIGT1-centred transcriptional cascade regulates nitrate signalling and incorporates phosphorus starvation signals in *Arabidopsis*

**DOI:** 10.1038/s41467-018-03832-6

**Published:** 2018-04-10

**Authors:** Yoshie Maeda, Mineko Konishi, Takatoshi Kiba, Yasuhito Sakuraba, Naoya Sawaki, Tomohiro Kurai, Yoshiaki Ueda, Hitoshi Sakakibara, Shuichi Yanagisawa

**Affiliations:** 1 Biotechnology Research Center, The University of Tokyo, Yayoi 1-1-1, Bunkyo-ku, Tokyo 113-8657 Japan; 2 RIKEN Center for Sustainable Resource Science, Suehiro 1-7-22, Tsurumi, Yokohama 230-0045 Japan

## Abstract

Nitrate is a nutrient signal that triggers complex regulation of transcriptional networks to modulate nutrient-dependent growth and development in plants. This includes time- and nitrate concentration-dependent regulation of nitrate-related gene expression. However, the underlying mechanisms remain poorly understood. Here we identify NIGT1 transcriptional repressors as negative regulators of the *Arabidopsis* *NRT2*.*1* nitrate transporter gene, and show antagonistic regulation by NLP primary transcription factors for nitrate signalling and the NLP-NIGT1 transcriptional cascade-mediated repression. This antagonistic regulation provides a resolution to the complexity of nitrate-induced transcriptional regulations. Genome-wide analysis reveals that this mechanism is applicable to *NRT2*.*1* and other genes involved in nitrate assimilation, hormone biosynthesis and transcription. Furthermore, the PHR1 master regulator of the phosphorus-starvation response also directly promotes expression of NIGT1 family genes, leading to reductions in nitrate uptake. NIGT1 repressors thus act in two transcriptional cascades, forming a direct link between phosphorus and nitrogen nutritional regulation.

## Abstract (web summary)

Plants respond to nutrients by modulating gene expression. Here, the authors show that nitrate suppresses *NRT2.1* nitrate transporter expression via NIGT1 transcriptional repressors and that phosphate starvation enhances this pathway via PHR1, thus linking phosphorus and nitrogen signalling.

## Introduction

Nitrogen (N) is a key plant macronutrient that is a major constituent of abundant essential compounds such as amino acids, nucleotides and chlorophyll^1^. N availability therefore has a profound effect on plant growth and productivity^1,2^. In order to thrive, plants adapt to fluctuations and differences in N availability in their environments through modulation of gene expression and metabolism. Nitrate in soils is the major N source for plants. Unlike microorganisms that use nitrate as an N source only in the absence of preferred energy-effective N sources such as ammonium and glutamine, higher plants take up and use nitrate to synthesise N-containing compounds even in the presence of other N sources^3^. Consistent with this preference, nitrate also serves as a signalling molecule that induces a variety of nitrate responses in plants^4,5^.

Modulation of plant gene expression by nitrate is complex. Nitrate provision immediately induces the expression of a wide variety of genes, including nitrate transport- and assimilation-associated genes, genes involved in phytohormone synthesis and response, and genes encoding regulatory proteins such as transcription factors and protein kinases^6,7^. Recent studies with *Arabidopsis* provide critical insights into the molecular mechanisms underlying nitrate-responsive gene expression. NIN-LIKE PROTEIN (NLP) transcriptional activators, which are highly conserved in plants, act as the primary transcription factors controlling the nitrate response^8,9^. Nitrate triggers Ca^2+^ signalling and activates calcium sensor kinases CPK10, CPK30 and CPK32. The CPKs phosphorylate NLPs at a conserved serine residue, and then phosphorylated NLPs accumulate in the nucleus and activate target genes^10^. The primary event in nitrate-induced transcriptional regulation is thus post-translational. Although the physiological functions of only two of the nine *Arabidopsis* NLPs (NLP7 and NLP8) are known, most NLPs are thought to be involved in the nitrate response^8,9,11–14^. Several additional transcription factor genes are induced by nitrate, and the encoded transcription factors are thought to evoke secondary transcriptional responses that activate or repress a new set of genes. Accordingly, these transcription factors produce a broad range of outcomes or modulate the expression of NLP targets by positive or negative feedforward mechanisms^15–18^. Although NLPs are known to directly regulate several nitrate-inducible genes through binding at specific *cis*-elements in the 5′ or 3′ flanking regions of the genes^9,14,19–21^, and nitrate-inducible transcription factors are known to be involved in the nitrate response^15,16,18^, transcriptional cascades involving NLPs have not been characterised. Thus, the molecular mechanisms underlying the complex regulation of gene expression during the nitrate response remain to be elucidated.

*Arabidopsis* *NRT2*.*1*, a typical nitrate-inducible gene, encodes a major high-affinity nitrate transporter that is indispensable for vigorous plant growth under nitrate concentrations in the natural ecosystem^22,23^. *NRT2*.*1* expression is rapidly and strongly induced upon provision of nitrate to N-starved or ammonium-cultivated *Arabidopsis* plants^24^. Upregulation is transient, and is suppressed within a few hours, suggesting that a time-dependent regulatory mechanism is also active^24^. Nitrate concentration also controls the steady-state levels of *NRT2*.*1* expression. Nitrate is most effective in promoting *NRT2*.*1* expression at low concentrations, with high concentrations (>0.5 mM) curbing the effect^24–26^. Similar responses were also reported for barley and *Nicotiana plumbaginifolia NRT2* genes^27,28^. Several factors participating in the regulation of *NRT2* expression were identified. For example, NLP7 binding at the *NRT2*.*1* promoter enhanced the expression^9^, TCP20 transcription factor also bound to the *NRT2*.*1* promoter^29^, and HNI9/AtIWS1 repressed expression of *Arabidopsis* *NRT2*.*1* under high N conditions through trimethylation of histone H3^30^. However, although the complex regulation of *NRT2* expression underpins its role in managing nitrate uptake in natural conditions^31^, the transcriptional repressor that modulates *NRT2* expression in a time- and nitrate concentration-dependent manner remains to be identified. Many other nitrate-inducible genes are also regulated in a time-dependent manner^16,32,33^, suggesting that an unknown regulatory mechanism might orchestrate the synchronous expression of these genes. However, such a mechanism is completely unknown.

Group II genes of the LBD gene family in *Arabidopsis* (*LBD37-39*) are potential negative regulators involved in time- and nitrate concentration-dependent modulations of nitrate-inducible gene expression^17^. *LBD37-39* are nitrate-inducible, and enhanced and reduced expression of several N metabolism-associated genes is seen with loss-of-function mutants and overexpression lines, respectively^17^. Another negative regulator candidate is NITRATE-INDUCIBLE GARP-TYPE TRANSCRIPTIONAL REPRESSOR1 (NIGT1). Rice NIGT1 (*Os*NIGT1) is a nitrate-inducible gene-encoded transcriptional repressor that binds to 5′-GAATC-3′ and 5′-GAATATTC-3′^16,34^. With the exception of *Os*NIGT1 targeting of Os*NIGT1* for negative feedback regulation, targets of *Os*NIGT1 remain to be identified; however, transgenic rice overexpressing Os*NIGT1* exhibits nitrate response-related phenotypes^16^. *Arabidopsis* possesses four homologues of *Os*NIGT1. The homologues of *Os*NIGT1 and three additional proteins with relatively lower similarities comprise the HRS/HHO family in *Arabidopsis*^35,36^. Although involvement of *Arabidopsis* homologues of *Os*NIGT1 in the phosphorus (P)-starvation response was suggested^35–37^, they are candidates for negative regulators for nitrate response due to their very strong induction by nitrate^7,36^. Intriguingly, N uptake was reduced by P limitation in several plant species^38–40^, probably to maintain the balance of N and P metabolism. In the light of the suggested involvement of *Arabidopsis* *Os*NIGT1 homologues in P-starvation response, they are also candidates for the regulators causing downregulation of nitrate uptake under P-starvation conditions.

To reveal the molecular mechanisms underlying complex regulation of gene expression during the nitrate response, we identify transcriptional repressors of the *NRT2*.*1* promoter in this study. The *NRT2*.*1* promoter is suppressed by *Arabidopsis* NIGT1 homologues, which are encoded by direct target genes of NLPs. Furthermore, expression of these genes is directly enhanced by the PHOSPHATE STARVATION RESPONSE 1 (PHR1) master regulator of P-starvation response^41,42^, inducing P-starvation-induced downregulation of nitrate uptake. These findings uncover two transcriptional cascades that govern complex expression of nitrate-inducible genes and for integration of N and P signalling.

## Results

### NIGT1.1 is a negative regulator of *NRT2*.*1*

**Fig. 1:**
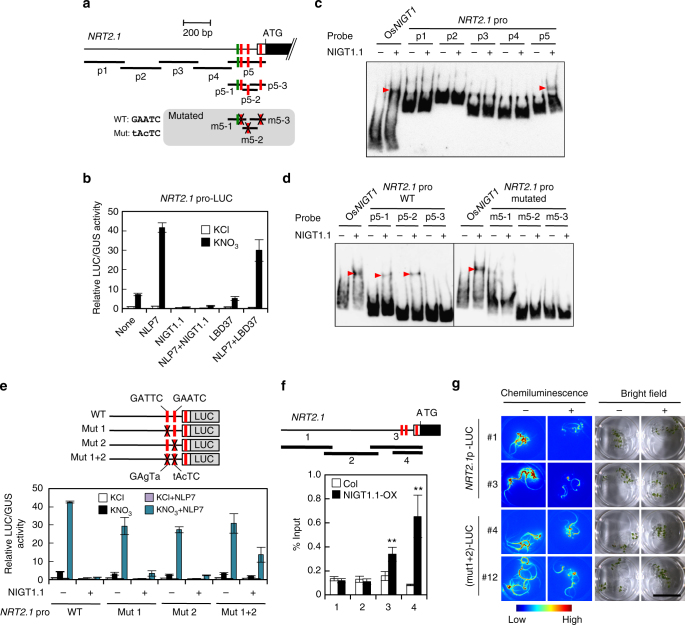
Repression of the *NRT2*.*1* promoter by NIGT1.1. **a** Structure of the *NRT2*.*1* promoter. Consensus sequences for NIGT1 binding (5′-GAATC-3′) and LBD binding (5′-GCGGCG-3′) are indicated by red and green bars, respectively. White and black boxes indicate the 5′ untranslated and coding regions, respectively. Probe DNAs used in EMSA (**c**, **d**) are shown below the promoter structure. To disrupt the NIGT1-binding sequences, mutated probes contained nucleotide substitutions, indicated by X. **b** Activation of the *NRT2*.*1* promoter by nitrate and NLP7 and repression by NIGT1.1 in protoplasts. Protoplasts co-transfected with the NLP7, NIGT1.1 or LBD37 expression vector or the empty vector (none) together with the reporter plasmid containing the *LUC* gene fused to the *NRT2*.*1* promoter were incubated in the presence of 1 mM KCl or KNO_3_. In **b** and **e**, LUC activity was normalised with GUS activity from the reference UBQ10-GUS plasmid and data are means ± s.d. of three biological replicates. **c**,** d** EMSA with recombinant NIGT1.1 protein and DNA probes from the *NRT2*.*1* promoter. Red arrowheads indicate positions of protein–DNA complexes caused by binding of NIGT1.1 to probe DNA. The Os*NIGT1* probe served as a positive control. **e** Effects of disruption of the NIGT1-binding sites on the *NRT2*.*1* promoter activity in protoplasts. Protoplasts co-transfected with the *LUC* gene fused to the wild-type or mutant *NRT2*.*1* promoter and the NLP7 expression vector or an empty vector were incubated in the presence of 1 mM KCl or KNO_3_. X indicates disrupted NIGT1 sites. **f** ChIP analysis of the *NRT2*.*1* promoter using Col and NIGT1.1-OX seedlings. Four regions were amplified by PCR with immunoprecipitated DNA. Data are means of four biological replicates with s.d. **p < 0.01 by one-tailed *t* test. **g** Effects of disruption of the NIGT1-binding sites on *NRT2*.*1* promoter activity in the presence of abundant N. Five seedlings of transgenic lines harbouring the *LUC* gene fused to the wild-type [*NRT2*.*1p*(WT)-*LUC*] or the mutated [(mut1+2)-LUC] *NRT2*.*1* promoter were incubated with (+) or without (−) 10 mM NH_4_NO_3_ for 24 h. Images of LUC activity in vivo and bright-field images were captured in two independent transgenic lines. Bar, 2 cm

Expression of *NRT2*.*1* is immediately repressed after activation in nitrate-treated *Arabidopsis* seedlings, suggesting that a transcriptional repressor might control repression. Scrutinisation of the *NRT2*.*1* promoter sequence revealed that it includes consensus sequences for NIGT1- (5′-GAATC-3′) and LBD-binding (5′-GCGGCG-3′)^43^ (Fig. 1a, Supplementary Fig. 1). To examine whether *Arabidopsis* homologues of *Os*NIGT1 and LBDs were negative regulators of *NRT2*.*1*, a reporter plasmid harbouring the luciferase reporter gene (*LUC*) under the control of the *NRT2*.*1* promoter was co-transfected into protoplasts alongside an effector plasmid for expression of LBD37 or an *Arabidopsis* homologue of *Os*NIGT1 (NIGT1.1). Protoplasts were prepared from N-starved *Arabidopsis* plants to allow detection of nitrate-responsive activation of the *NRT2*.*1* promoter caused by endogenous NLPs^20^. Nitrate-induced activation was detected when an empty vector was used as a control effector plasmid, and NLP7 expression enhanced nitrate-responsive activation of the *NRT2*.*1* promoter as reported previously^9^ (Fig. 1b). Nitrate-dependent activation and NLP7-induced enhancement were both suppressed by co-expression of NIGT1.1, whereas co-expression of LBD37 had almost no impact on *NRT2*.*1* promoter activity. This indicated that NIGT1.1 had a specific negative effect on the *NRT2*.*1* promoter and also that NIGT1 could override the NLP7-driven activation of *NRT2*.*1* expression in response to nitrate (Fig. 1b).

To examine the role of *Arabidopsis* NIGT1s in controlling *NRT2*.*1* expression, NIGT1-binding sites in the *NRT2*.*1* promoter were investigated using an electrophoretic mobility shift assay (EMSA) with recombinant NIGT1.1 protein (Fig. 1c, d). NIGT1.1 bound specifically to probe p5 (−217 to +63, relative to the transcription start site) (Fig. 1c), which contained three copies of the putative NIGT1 site (Fig. 1a), and we therefore examined the effects of mutations in these sites (conversion of 5′-GAATC-3′ to 5′-tAcTC-3′). The results indicated that NIGT1.1 bound to the first and second sites in the proximal region of the *NRT2*.*1* promoter but not to the third one (Fig. 1d). Consistent results were obtained in co-transfection experiments with reporter plasmids containing *LUC* under the control of mutated *NRT2*.*1* promoters harbouring the same mutations (Fig. 1e). Simultaneous disruptions of the first and second GAATC sequences significantly abolished NIGT1.1-dependent repression (Fig. 1e), while disruptions of the third one did not affect NIGT1.1-dependent repression (Supplementary Fig. 2). Thus, NIGT1.1 repressed the *NRT2*.*1* promoter through interactions at these two sites. Any mutations on the NIGT1-binding sites did not affect NLP7-induced activation of the *NRT2*.*1* promoter (Fig. 1e, Supplementary Fig. 2). We therefore hypothesised that NLP7 and NIGT1.1 independently regulated the *NRT2*.*1* promoter. To confirm this, we identified NLP-binding sites in the *NRT2*.*1* promoter. We sought for putative NLP-binding sites in two regions homologous between the *NRT2*.*1* and *NRT2*.*2* promoter sequences, because *NRT2*.*2*, another high-affinity nitrate transporter gene neigbouring *NRT2*.*1*, very resembles *NRT2*.*1* in both expression pattern and structure^22,44^. Since two putative NLP-binding sites (−714 to −707 and −172 to −150) were found in the homologous regions (Supplementary Fig. 1), EMSA were performed with DNA probes containing these sites. Consequently, NLP7 was shown to bind to both sites (Supplementary Fig. 3a,b). Co-transfection assays consistently indicated that the disruption of either site reduced NLP7-dependent transactivation and also that the disruption of both sites almost diminished both nitrate-inducible activation and NLP7-dependent transactivation (Supplementary Fig. 3c−e). Furthermore, the disruption of these sites did not affect the NIGT1.1-induced repression (Supplementary Fig. 3f), indicating that NLP7 and NIGT1.1 independently regulate the *NRT2*.*1* promoter through interactions with distinct sites.

The binding of NIGT1.1 to the *NRT2*.*1* promoter in planta was also corroborated using transgenic *Arabidopsis* plants overexpressing MYC-tagged NIGT1.1 (NIGT1.1-OX lines) (Supplementary Fig. 4). Chromatin immunoprecipitation (ChIP) analysis using the NIGT1.1-OX line revealed that NIGT1.1 specifically bound to the proximal promoter region containing the identified NIGT1-binding sites (Fig. 1f). NIGT1-binding site-dependent repression of the *NRT2*.*1* promoter was further characterised using in vivo imaging of LUC activity. We introduced *LUC* under the control of wild-type and mutated *NRT2*.*1* promoters into the wild-type *Arabidopsis* and examined LUC activity in seedlings of the obtained transgenic lines (Fig. 1g). LUC production decreased upon N supplementation (ammonium nitrate) with both wild-type and mutated promoters, but the decrease was far weaker with the mutated promoter, indicating that NIGT1 binding at these sites was associated with repression of *NRT2*.*1* promoter activity. In vivo imaging also clarified concentration-dependent nitrate induction of the *NRT2*.*1* promoter and also suggested that NIGT1-mediated repression is evident in the presence of high concentrations of nitrate (Supplementary Fig. 5). Together, these results indicate that NIGT1.1 acts as a negative regulator of *NRT2*.*1* and competes with positive regulation by NLPs.

### Redundancies of NIGT1*-*clade genes in *Arabidopsis*

**Fig. 2:**
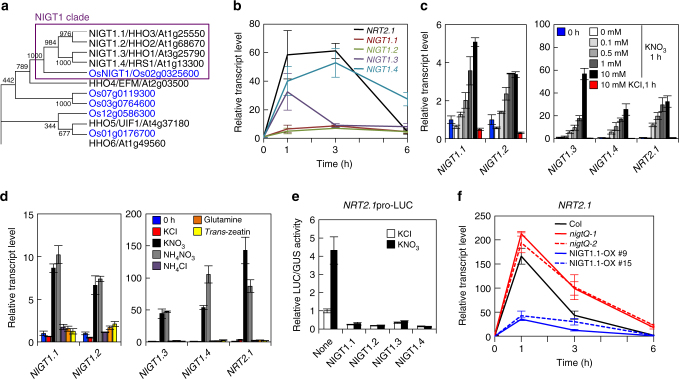
NIGT1 proteins redundantly regulate *NRT2*.*1* expression. **a** Phylogenic analysis of *Arabidopsis* and rice NIGT1/HHO proteins. *Arabidopsis* and rice proteins are shown in black and blue, respectively. Numbers indicate bootstrap values of 1000 iterations. **b** Time-dependent expression patterns of NIGT1-clade genes after 10 mM KNO_3_ supply. **c** Concentration-dependent induction by nitrate. RNA was prepared from ammonium-grown seedlings before treatment and after treatment with 0, 0.1, 0.5, 1 and 10 mM KNO_3_ for 1 h. A 10 mM KCl treatment was used as a control. **d** Nitrate-specific induction of NIGT1*-*clade genes. Ammonium-grown seedlings were treated for 1 h with 10 mM of each chemical, except for *trans-*zeatin (5 µM). **e** Repression of the *NRT2*.*1* promoter by NIGT1 proteins. Protoplasts co-transfected with NIGT1 expression vectors or the empty vector (none) together with the reporter plasmid containing the *LUC* gene under the control of the *NRT2*.*1* promoter were incubated in the presence of 1 mM KCl or KNO_3_. LUC activity was normalised to GUS activity from the co-transfected UBQ10-GUS plasmid. Data are means ± s.d. of three biological replicates. **f** Transcript levels of *NRT2*.*1* in the *nigt1* quadruple mutants (*Q-1* and *Q-2*) and NIGT1.1-OX plants after 10 mM KNO_3_ supply. Values are normalised to those of *UBQ10*, and are means of biological triplicates ± s.d. (**b**, **c**, **d**, **f**)

Phylogenic analysis indicated a particularly close relationship between *Os*NIGT1 and four *Arabidopsis* proteins of the HRS/HHO family^17,37,38^ as demonstrated by very high bootstrap values (Fig. 2a). *Os*NIGT1 and these four proteins were distinguishable from others of the HRS/HHO family and formed a separate clade^16,35,36^, referred to hereafter as the NIGT1 clade (Fig. 2a). Previous microarray analysis showed that all of the *Arabidopsis* NIGT1-clade genes (*NIGT1*.*1/HHO3*, *NIGT1*.*2/HHO2*, *NIGT1*.*3/HHO1* and *NIGT1*.*4/HRS1*) were induced by nitrate treatment^7^. We therefore examined whether these genes exhibited similar expression patterns and whether their roles were redundant. RT-quantitative real-time PCR (qPCR) analysis was used to characterise the temporal and nitrate concentration-dependent expression of these genes (Fig. 2b, c). Nitrate treatment of nitrate-starved seedlings induced expression of NIGT1-clade genes; however, no induction was seen with ammonium, glutamine or *trans*-zeatin (tZ), a type of cytokinin (N response-related phytohormone) (Fig. 2d). These expression patterns were similar to those of Os*NIGT1*^16^ and *NRT2*.*1*. This suggested that all NIGT1-clade genes were primary nitrate-inducible genes, like *NRT2*.*1*. However, *NIGT1*.*1* and *NIGT1*.*2* induction was less pronounced than induction of *NIGT1*.*3* and *NIGT1*.*4* (Fig. 2b−d), and *NIGT1*.*1* and *NIGT1*.*2* were expressed in both shoots and roots in contrast with the root-specific expression of *NIGT1*.*3* and *NIGT1*.*4* (Supplementary Fig. 6a).

NLPs direct the majority of primary nitrate-induced gene expression^8,9^. Our transcriptome analysis with transgenic *Arabidopsis* in which NLP activity was reduced by the expression of a chimera repressor NLP6-SUPRD (NLP6-SUPRD lines)^21^ also suggested that nitrate-inducible expression of NIGT1-clade genes was under the control of NLP activity^21^. We thus performed co-transfection assays to examine whether NLP7 activated the promoters of NIGT1-clade genes (Supplementary Fig. 7). The 1 kb promoters of *NIGT1*.*1*, *NIGT1*.*3* and *NIGT1*.*4* were activated by co-expression of NLP7 in the presence of nitrate, suggesting the existence of the NLP-NIGT1 transcriptional cascade. Neither nitrate- nor NLP7-dependent activation of the *NIGT1*.*2* promoter was observed in this assay, suggesting that nitrate-responsive *cis*-elements for *NIGT1*.*2* may be outside the promoter fragment used. Co-transfection experiments also showed that all *Arabidopsis* NIGT1s were transcriptional repressors of the *NRT2*.*1* promoter (Fig. 2e). This suggests that *Arabidopsis* NIGT1s have a redundant role in regulating *NRT2*.*1* expression.

Time-course analysis of expression of the NIGT1-clade genes suggested that NLP-NIGT1 transcriptional cascade-mediated suppression of *NRT2*.*1* occurred after activation by NLPs. This antagonistic relationship may be involved in downregulation of *NRT2*.*1* expression after a transient peak induced by nitrate treatment. We therefore analysed the time-dependent expression pattern of *NRT2*.*1* in *nigt1*.*1 nigt1*.*2 nigt1*.*3 nigt1*.*4* quadruple mutants (*nigtQ-1* and *Q-2*) and NIGT1.1-OX lines (Fig. 2f). A transient *NRT2*.*1* expression peak was observed in all lines, but expression levels were higher in the quadruple mutants and lower in the two independent NIGT1.1-OX lines. Furthermore, modified levels of *NRT2*.*1* transcripts in the single, double, triple and quadruple *nigt1* mutants suggested that all *NIGT1*-clade genes are involved in the regulation of *NRT2*.*1* expression (Supplementary Fig. 8c), consistent with the expression of NIGT1-clade genes in outer cell layers in roots (Supplementary Fig. 6c) where *NRT2*.*1* is expressed^25,26,45^. The relatively higher expression level of *NIGT1*.*4* in cortex and root hair-forming cells^35^ (Supplementary Fig. 6b) might correlate with the stronger effect of the *nigt1*.*4* mutation on *NRT2*.*1* expression (Supplementary Fig. 8c).

### Genome-wide survey of target genes of the NLP-NIGT1 cascade

**Fig. 3:**
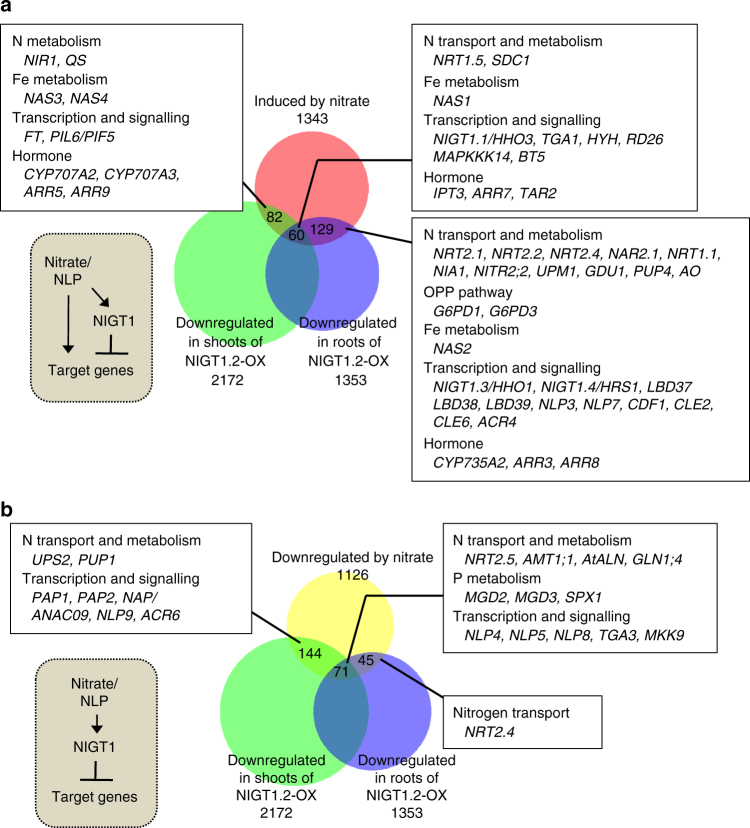
Nitrate-responsive and NIGT1-regulated genes. **a** A Venn diagrams showing an overlap between genes upregulated by nitrate after 30 min or 3 h and genes downregulated in NIGT1.2-overexpressing plants. **b** A Venn diagram showing an overlap between genes downregulated by nitrate after 30 min or 3 h and downregulated in NIGT1.2-overexpressing plants. Numbers of genes are shown. Note that *NRT2*.*4* was upregulated at 30 min after nitrate treatment but downregulated at 3 h

**Fig. 4:**
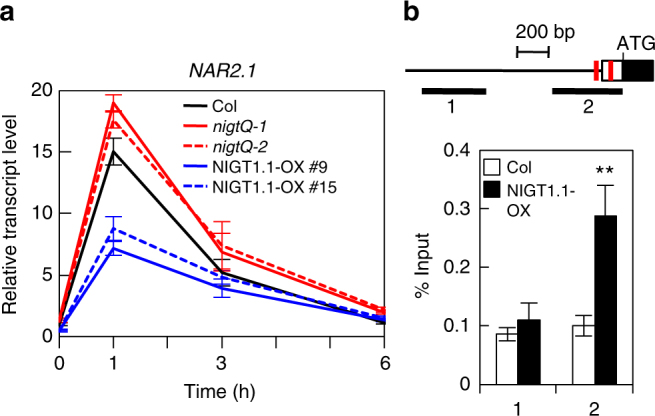
Repression of *NAR2*.*1* by NIGT1s. **a** Time-dependent expression of *NAR2*.*1* in *Arabidopsis* Col, *nigt1* quadruple mutants (*Q-1* and *Q-2*), and two NIGT1.1-OX lines (#9 and #15). Seedlings were harvested at the indicated time points after supply of 10 mM KNO_3_. Values are normalised with expression levels of *UBQ10*, and means of biological triplicates ± s.d. are shown. **b** Binding of NIGT1.1 to the *NAR2*.*1* promoter in vivo. ChIP analysis was carried out with Col and NIGT1.1-OX seedlings. Two different regions of the *NAR2*.*1* promoter that contained or did not contain putative NIGT1-recognition sequences (red bars) were amplified from immunoprecipitated DNA by PCR. Data are means of four biological replicates with s.d. **p < 0.01 by one-tailed *t*-test compared with corresponding values obtained with Col seedlings

**Fig. 5:**
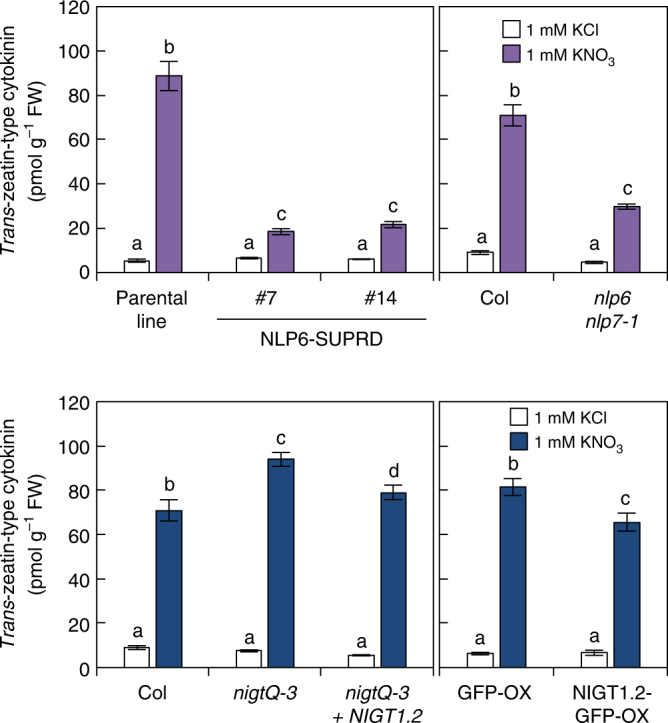
NLP and NIGT1 activity affects tZ-type cytokinin content. Cytokinin contents in the seedlings treated with 1 mM KNO_3_ or KCl for 24 h were measured. The cytokinin contents in two NLP6-SUPRD lines were compared with those in its parental line (4xNRE-35Smin-GUS)^21^, and the cytokinin contents in the *nlp6 nlp7-1* mutant, the *nigt1* quadruple mutant (*nigtQ-3*), and the complementation line of *nigtQ-3*, which was produced by expression of *NIGT1*.*2*, were compared with those in Col seedlings. The cytokinin contents in the NIGT1 overexpressor (NIGT1.2-GFP-OX), which was produced by the overexpression of NIGT1.2 fused to GFP, were compared with those in the GFP overexpressor line (GFP-OX). Values are mean ± s.d. (*n* = 4~5). Different letters denote significant difference by Tukey’s HSD (*p* < 0.05)

Time-dependent expression control after nitrate supply is not specific to *NRT2* genes but is common to many nitrate-inducible genes^16,32,33^. To investigate whether antagonistic regulation by NLP and the NLP-NIGT1 cascade modulated the expression of other genes, a genome-wide survey of the target genes of the NLP-NIGT1 cascade was performed using previous transcriptome data of nitrate-responsive genes^7^ and genes that were downregulated in *Arabidopsis* NIGT1.2 overexpressors (accession number GSE100903 in Gene Expression Omnibus)^46^ (Fig. 3, Supplementary Data 1 and 2). The combined survey revealed that out of 1343 genes induced by nitrate after 30 min or 3 h^7^, 271 genes (20%) were repressed by constitutive overexpression of NIGT1.2 (Fig. 3a). Lower expression levels of these genes in the NLP6-SUPRD lines^21^ were confirmed, suggesting that these genes are under the control of NLP activity (Supplementary Data 1). Therefore, these genes are likely under the control of both NLP-mediated activation and NLP-NIGT1 transcriptional cascade-mediated repression, and expression of these genes may thereby transiently increase after nitrate supply. The gene list includes not only N transport and assimilation-associated genes (*NRT2*.*1*, *NRT2*.*2*, *NAR2*.*1*, etc.), but also genes involved in iron metabolism, the oxidative pentose phosphate (OPP) pathway for supplying reducing power for N assimilation, transcription (*TGA1*^15^ and *HYH*), and hormone metabolism (*IPT3*, *CYP735A2*^47^, *TAR2*, and *CYP707A2*,*3*^14^) (Fig. 3a, Supplementary Data 1). Like *NRT2*.*1*, *NAR2*.*1* (also called *NRT3*.*1*), which encodes an accessory protein essential for NRT2.1 activity^45,48^, exhibited a time-dependent expression pattern that was modulated in *nigtQ* mutants and NIGT1.1-OX lines (Fig. 4a). Furthermore, ChIP assay with the NIGT1.1-OX line suggested that NIGT1 bound specifically to a region of the *NAR2*.*1* promoter that contained consensus sequences for NIGT1 binding (Fig. 4b). This suggests that the molecular mechanism consisting of nitrate signalling, NLPs, and the NIGT1 transcriptional cascade coordinately modulates expression of *NRT2*.*1* and *NAR2*.*1* to control nitrate uptake activity in response to the abundance of environmental nitrate. *CYP735A2* and *HYH* were also identified as possible targets of NLPs and the NLP-NIGT1 cascade (Fig. 3a). *CYP735A2* encodes cytokinin hydroxylase, which catalyses the biosynthesis of tZ-type cytokinin, and *HYH* encodes a bZIP transcription factor involved in light-mediated development. Expression of these genes was similarly downregulated in NIGT1.1-OX plants, and NIGT1.1 specifically bound to the regions of their promoters containing NIGT1-recognition sequences in vivo (Supplementary Fig. 9). Hence, antagonistic regulation by NLPs and the NLP-NIGT1 cascade likely modulates the expression of a variety of genes associated with the nitrate response. For further investigation of physiological relevance of the NLP-NIGT1 cascade, we focused on *CYP735A2*, because this gene encodes a key enzyme for biosynthesis of tZ-type cytokinin that is intimately associated with nitrate response^47,49^. We measured the tZ content in the NLP6-SUPRD line, the *nlp6 nlp7-1* double mutant, the *nigt1* quadruple mutant, and the NIGT1.2 overexpressor line (Fig. 5, Supplementary Table 1). Supply of nitrate strongly increased the tZ content in the N-starved wild-type seedlings, and this increase was impaired in the NLP6-SUPRD and *nlp6 nlp7-1* lines. On the other hand, the quadruple *nigt1* mutation enhanced the nitrate-induced increase in the tZ content, whereas the overexpression of NIGT1.2 alleviated it. These results revealed a role of NIGT1 proteins in modulating the NLP-mediated physiological response.

Our genome-wide survey also provided a list of genes whose expression was reduced by both nitrate treatment and NIGT1 overexpression (Fig. 3b, Supplementary Data 2). These genes appear to be regulated by the NLP-NIGT1 cascade alone, and thereby they are candidates for the genes whose expression is negatively regulated by nitrate signalling. Of 1126 genes downregulated by nitrate treatment, 260 genes were downregulated in the NIGT1.2 overexpressor, including genes encoding transporters for nitrate, ammonium and ureide, and transcription factors.

### Autoregulation of NIGT1-clade genes

**Fig. 6:**
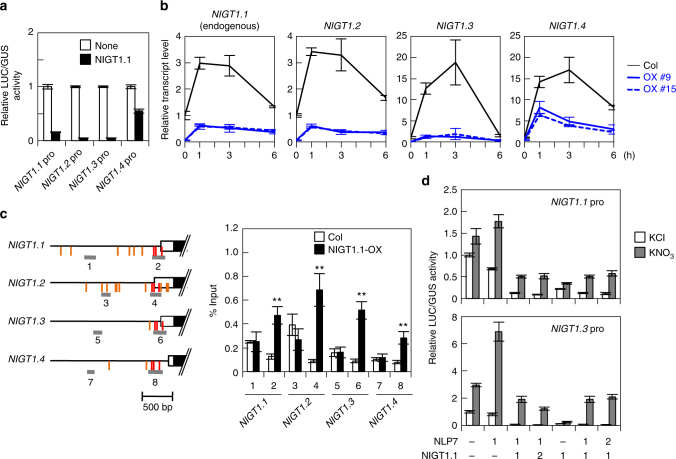
Autoregulation of NIGT1-clade genes. **a** Repression of NIGT1-clade gene promoters by NIGT1.1. A reporter plasmid that contained the *LUC* gene under the control of an *NIGT1* promoter was co-transfected in protoplasts alongside the NIGT1.1 expression vector and the UBQ10-GUS reference plasmid. LUC activity was normalised with GUS activity. Data are means ± s.d. of three biological replicates. **b** Reduced expression of endogenous *NIGT1*.*1* as well as *NIGT1*.*2*, *NIGT1*.*3* and *NIGT1*.*4* in NIGT1.1-OX lines. Ammonium-grown *Arabidopsis* Col and NIGT1.1-OX seedlings were treated with 10 mM KNO_3_ for the indicated periods and used for RT-qPCR analysis. Values are normalised to *UBQ10* expression levels, and means of biological triplicates with s.d. are shown. **c** Binding of NIGT1.1 to the *NIGT1* gene promoters in vivo. ChIP analysis was performed with Col and NIGT1.1-OX seedlings. Two different promoter regions were amplified from immunoprecipitated DNA. Data are means of four biological replicates with s.d. **p < 0.01 by one-tailed *t* test compared with corresponding values obtained with Col seedlings. Positions of the conserved and nonconserved putative NIGT1-binding sites are shown by vertical red and orange lines, respectively. **d** Antagonistic regulation of NIGT1-clade gene promoters by NLP7 and NIGT1.1. Expression vectors for NLP7 and NIGT1.1 were co-transfected into protoplasts at different ratios, together with a reporter plasmid containing the *LUC* gene under the control of the *NIGT1*.*1* or *NIGT1*.*3* promoter. Transfected protoplasts were incubated in the presence of 10 mM KCl or KNO_3_. LUC activity was normalised with GUS activity, and relative LUC activities are means ± s.d. of three biological replicates

Our genome-wide survey suggested that NIGT1-clade genes might themselves be targets of the NLP-NIGT1 cascade (Fig. 3a). This is consistent with the previous evidence suggesting that OsNIGT1 is autoregulated^16^. Co-transfection assays were used to determine whether NIGT1-clade genes in *Arabidopsis* were autoregulated (Fig. 6a). NIGT1.1 suppressed the promoters of all the NIGT1-clade genes. Furthermore, in transgenic *Arabidopsis* plants constitutively overexpressing NIGT1.1, *NIGT1*.*2-1*.*4* and endogenous *NIGT1*.*1* exhibited reduced levels of time-dependent gene expression in both the presence and absence of nitrate (Fig. 6b). In a ChIP assay using the NIGT1.1-OX line, regions that contained putative NIGT1-binding sites conserved in *Arabidopsis* NIGT1*-*clade genes and Os*NIGT1*, but not in other *HHO* genes^16^ (regions 2, 4, 6 and 8), specifically co-immunoprecipitated with NIGT1.1, suggesting that NIGT1s bound specifically to these conserved sites in vivo (Fig. 6c). Co-expression of NIGT1.1 also decreased both nitrate-induced activation and NLP-dependent transactivation of the *NIGT1*.*1* and *NIGT1*.*3* promoters (Fig. 6d). These results suggest that feedback regulation of NIGT1-clade genes is mediated by NIGT1 transcriptional repressors, indicating that nitrate signalling is complex and is driven by the integration of the NLP-NIGT1 cascade and the NIGT1 autoregulation.

### PHR1 activates the promoters of NIGT1-clade genes

**Fig. 7:**
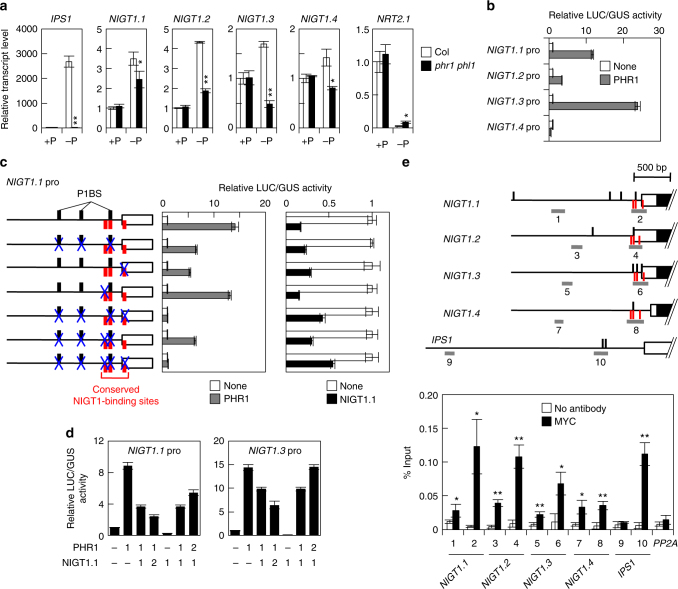
PHR1-dependent activation of NIGT1-clade genes. **a** Effects of P starvation on the expression of NIGT1-clade genes and *NRT2*.*1* in roots of *Arabidopsis* Col and *phr1 phl1* double mutant. Roots of *Arabidopsis* plants that were grown hydroponically under P-sufficient (+P) or P-starved (−P) conditions for 5 days were used for RT-qPCR analysis. *IPS1* is a positive control for P starvation. Values are normalised with expression levels of *PEX4*, and means of biological triplicates with s.d. are shown. *p < 0.05, **p < 0.01 by two-tailed *t*-test compared with corresponding Col values. **b** Transactivation of NIGT1 promoters by PHR1. A reporter plasmid containing 1 kb promoter fragments of NIGT1*-*clade genes fused to the *LUC* gene was co-transfected into protoplasts with the PHR1 expression vector or an empty vector (none) and the UBQ10-GUS plasmid. **c** Identification of sites mediating PHR1 regulation and autoregulation in the *NIGT1*.*1* promoter. Transactivation of wild-type and mutated *NIGT1*.*1* promoters by PHR1 and repression by NIGT1.1 were analysed by co-transfection assays. The horizontal line indicates the 1 kb *NIGT1*.*1* promoter. Vertical black and red lines indicate the P1BS sequences and the conserved NIGT1 sites, respectively. Mutated sites are indicated with blue X. **d** Competitive regulation of the NIGT1-clade gene promoters by PHR1 and NIGT1. Expression vectors for PHR1 and NIGT1.1 were co-transfected into protoplasts at different ratios, together with the *LUC* reporter gene under the control of the *NIGT1*.*1* or *NIGT1*.*3* promoter. In **b**−**d**, relative LUC activity is given as mean ± s.d. of three biological replicates. **e** Binding of PHR1 to the NIGT1-clade gene promoters in vivo. Two different regions from each NIGT1-clade gene promoter were amplified in a ChIP assay of transgenic plants expressing MYC-tagged PHR1 cultured under the P-starved condition. The *IPS1* promoter is a known target of PHR1 and is used as a positive control, and *PP2A* is a negative control. Data are means of three biological samples and shown with s.d. *p < 0.05, **p < 0.01 by one-tailed paired *t*-test compared with corresponding control (no antibody) values

Nitrate uptake is repressed by P starvation^38,39,50^. Although expression of NIGT1*-*clade genes is strongly induced by nitrate, several previous studies suggested that the NIGT1-clade genes might also be involved in the P-starvation response^35–37^. Supporting this, DNA microarray data provided previously by Bustos et al.^42^ suggested that expression of *NIGT1*.*1*, *1*.*2* and *1*.*3* likely decreased in an *Arabidopsis* *phr1 phl1* mutant. PHR1 and its close homologue, PHR1-LIKE1 (PHL1), are key transcriptional activators that recognise the 5′-GNATATNC-3′ sequence (called P1BS)^41^ and orchestrate P-starvation-induced gene expression^42^. PHR1, PHL1 and NIGT1s belong to the GARP family of plant transcription factors, and the P1BS sequence overlaps with one of the NIGT1-recognition sequences (5′-GAATATTC-3′)^16,34^. Thus, we hypothesised that PHR1 and PHL1 might directly activate expression of NIGT1-clade genes through binding to NIGT1-binding sites and other P1BS sites to downregulate *NRT2*.*1* expression. To test this hypothesis, we initially confirmed the effects of P starvation and *phr1 phl1* double mutation on the expression of NIGT1*-*clade genes using RT-qPCR analysis. P starvation upregulated the expression of all NIGT1-clade genes, and this upregulation was compromised in the *phr1 phl1* mutant (Fig. 7a). *NRT2*.*1* expression was strongly downregulated under the P-starved conditions, but downregulation was alleviated in *phr1 phl1*. Furthermore, co-transfection assays with the PHR1 expression vector revealed that PHR1 activated the *NIGT1*.*1*, *NIGT1*.*2* and *NIGT1*.*3* (but not *NIGT1*.*4*) promoters in vivo (Fig. 7b). Next, the sites mediating PHR1-dependent transactivation in the *NIGT1*.*1* promoter were determined using mutational analysis. Three copies of P1BS are found within the *NIGT1*.*1* promoter, one of which is also a conserved NIGT1-binding site (Fig. 7c)^16^. The disruption of each P1BS slightly decreased activation by PHR1 (Supplementary Fig. 10). Simultaneous disruption of all three copies of P1BS reduced the promoter activity by half compared with the wild-type promoter (Fig. 7c, Supplementary Fig. 10). Unexpectedly, disruption of the proximal NIGT1-binding sequence (5′-GATTC-3′) also reduced promoter activity to half that of the wild-type promoter (Fig. 7c), and the simultaneous disruption of this GATTC sequence and all three copies of P1BS mostly abolished activation by PHR1 (Fig. 7c), indicating that these sites were recognised by PHR1. Co-transfection assays also revealed that, as with the combination of NLP7 and NIGT1s, transactivation caused by PHR1 was suppressed by co-expression of NIGT1s, indicating competition between PHR1-dependent activation and NIGT1-dependent repression on the expression of NIGT1-clade genes in vivo (Fig. 7d). Furthermore, ChIP assays with transgenic *Arabidopsis* plants overexpressing PHR1 suggested that PHR1 bound to the proximal region of the *NIGT1*.*1* promoter and other NIGT1-clade gene promoters in vivo (Fig. 7e). Slight amplification of regions 1, 3, 5 and 7 was probably because sonication in the ChIP procedure causes inhomogeneous fragmentation of DNA. These results indicated the existence of the PHR1-NIGT1 transcriptional cascade. Although the identified PHR1-binding sites in proximal regions were conserved in all *NIGT1* promoters, the recovery of the corresponding region of the *NIGT1*.*4* promoter (region 8) was lower than in other NIGT1-clade gene promoters (regions 2, 4 and 6) and very similar to the value of a region not containing P1BS (region 7) in the ChIP assays. This was consistent with relatively lower induction of *NIGT1*.*4* expression and no transactivation of the *NIGT1*.*4* promoter by PHR1. Because not all P1BS motifs are functional for P-starvation response^42^, the sequences surrounding the P1BS motif in the *NIGT1*.*4* promoter might affect its functionality.

### The role of the PHR1-NIGT1 cascade in nitrate uptake

**Fig. 8:**
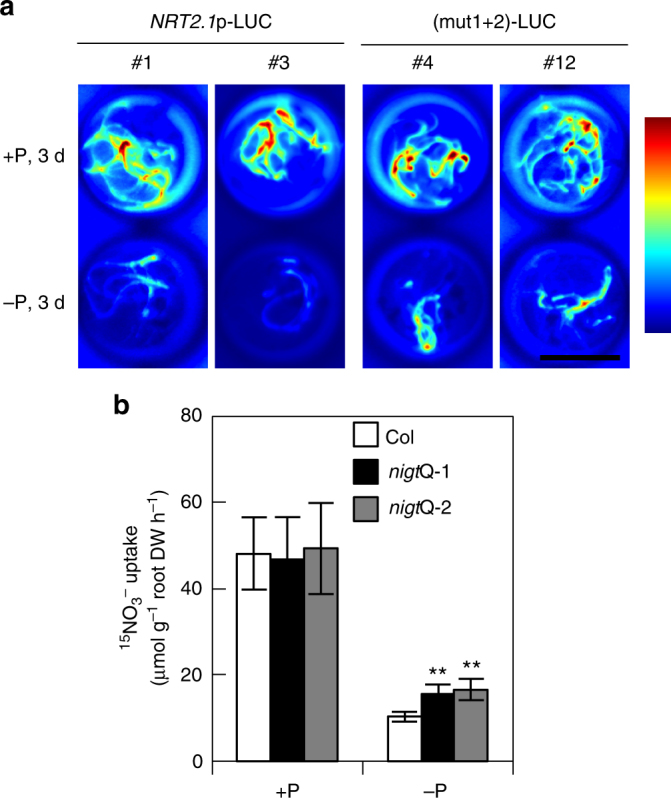
The PHR1-NIGT1 cascade modulates nitrate uptake. **a** NIGT1-binding site-mediated P-starvation response of *NRT2*.*1* promoter activity. Transgenic *Arabidopsis* seedlings harbouring the *LUC* gene under the control of the wild-type or (mut1+2) mutant *NRT2*.*1* promoter were grown under P-sufficient (+P) or P-starved (−P) conditions for 3 days and then treated with 1 mM luciferin. Images of LUC activity in living seedlings were captured in two independent transgenic lines. Scale bar, 1 cm. **b** Nitrate uptake activity of *nigt1* quadruple mutants (*Q-1* and *Q-2*) during P starvation. ^15^NO_3_^−^ uptake by *Arabidopsis* Col and mutant seedlings grown hydroponically under P-sufficient (+P) or P-starved (−P) conditions for 5 days was measured at 0.2 mM K^15^NO_3_. Values are means of five biological replicates ± s.d. **p < 0.01 by two-tailed *t*-test

PHR1-NIGT1 cascade-dependent regulation of the *NRT2*.*1* promoter activity was investigated by in vivo imaging of LUC activity in transgenic plants harbouring *LUC* fused to the wild-type promoter or a mutant promoter with disrupted NIGT1-binding sites [mut(1+2) promoter in Fig. 1e]. Levels of LUC activity driven by the wild-type and mutant *NRT2*.*1* promoters were similar under the P-sufficient condition, and LUC levels decreased in both after P-starvation treatment for 3 days. However, LUC levels were higher with the mutant than with the wild type under P-deficient conditions (Fig. 8a), indicating that NIGT1-binding sites were partly involved in P-starvation-induced repression of the *NRT2*.*1* promoter. Because NRT2.1 is the high-affinity nitrate transporter that plays the major role in uptake of nitrate from soils, we next examined nitrate uptake activity of wild-type plants and *nigt1* quadruple mutants grown with or without P (Fig. 8b). Nitrate uptake was comparable between wild-type plants and *nigt1* quadruple mutants under the P-sufficient condition. Nitrate uptake decreased in P-starved seedlings, but uptake was higher in the *nigt1* quadruple mutant than in the wild type under the P-deficient condition (Fig. 8b). These results suggest that P starvation partly influences nitrate uptake through the PHR1-NIGT1-NRT2.1 transcriptional cascade.

## Discussion

In the current study, we identified the NLP-NIGT1 and PHR1-NIGT cascades, both of which downregulate *NRT2*.*1* in opposition to direct activation by NLPs. These findings demonstrate the molecular mechanisms underlying the complex regulation of the expression of nitrate-inducible genes.

**Fig. 9:**
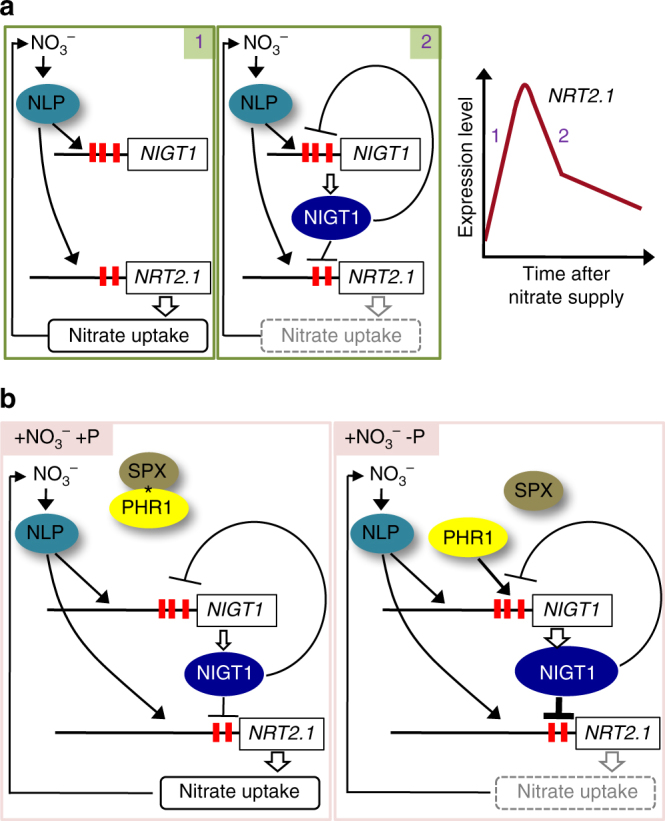
Model for the NIGT1-centred transcriptional cascade. **a** Model for the transcriptional cascade that modulates nitrate uptake in response to fluctuations in the nitrate condition. Nitrate activates NLP, which directly upregulates both the *NRT2*.*1* and NIGT1-clade gene promoters. Thus, *NRT2*.*1* expression is initially activated (1) and then repressed by expression of NIGT1*-*clade genes (2). Increases in *NRT2*.*1* expression lead to enhanced uptake of nitrate as a substrate for nitrate assimilation and also a signal for activating NLP. The expression level of *NRT2*.*1* is modulated by the balance of these positive and negative regulatory loops. **b** Model for the transcriptional cascade that integrates nitrate and P-starvation signalling pathways. When P nutrient (phosphate) is abundant, PHR1 is kept inactive by the SPX–inositol polyphosphate (*) complex. Thus, the nitrate-NLP cascade activates the *NRT2*.*1* promoter independently of the PHR1 pathway. Upon P depletion, PHR1 is freed and enhances the expression of NIGT1-clade genes, leading to reductions in both *NRT2*.*1* expression and nitrate uptake

The identification of the NLP-NIGT1 cascade allows us to propose a simple model to explain the transiently high expression and subsequent downregulation that are very frequently exhibited by primary nitrate-inducible genes after nitrate treatment (Fig. 9a). In this model, nitrate-activated NLPs at first rapidly maximise the expression levels of their targets and then expression is downregulated by NLP-induced NIGT1s. Thus, the transcript levels of these genes are determined by the balance of activated forms of NLPs and NIGT1s. Although we demonstrated antagonistic regulation by NLP7 and NIGT1s only at the *NRT2*.*1* promoter, the same mechanism likely applies to downregulation of other nitrate-inducible genes, including N metabolism- and hormone-related genes and transcription factor genes for the secondary nitrate response (Fig. 3). In fact, we showed that nitrate-induced increases in the cytokinin content were modified under the control of NLP and NIGT1 activities (Fig. 5, Supplementary Table 1). Hence, NIGT1 proteins act as modulators for nitrate-responsive transcription, and the antagonistic regulation by NLPs and the NLP-NIGT1 cascade may be critical to determining the capacity of N transport and assimilation and other nitrate responses. We propose that the NLP-NIGT1 cascade is one of critical modulators for nitrate-responsive transcription on the basis of our findings; however, other factors may act to regulate the temporal expression patterns of nitrate-inducible genes including *NRT2*.*1*, because the mutated *NRT2*.*1* promoter with the disruptions of NIGT1-biding sites was still repressed during nitrate treatment. One of such factors may be the nitrate-induced accumulation of N-metabolites and cytokinin that repress the expression of nitrate-inducible genes, including *NRT2*.*1*^25,26,49,51^. Another is degradation of NLPs, because NLPs in the active form were more rapidly degraded, compared with ones in the inactive form (Supplementary Fig. 11).

Antagonistic regulation by NLPs and NIGT1s may explain not only transient changes in nitrate-inducible expression after nitrate supply but also different expression levels at the steady state under different N concentrations. As reported previously, *NRT2*.*1* expression is high at appropriate concentrations of nitrate but is lower at higher concentrations of nitrate^24–26^. This may be partly mediated by N-metabolites^25,26,49^, but, as the phenomenon is observed even in the presence of ammonium, nitrate itself is a key determinant^31^. When the nitrate concentration is higher than the appropriate concentration range, higher amounts of NIGT1s may accumulate and suppression by NIGT1s may dominate. Consistent with this hypothesis, two NIGT1-binding sites are located in the 150 bp promoter region of the *NRT2*.*1* promoter that was previously shown to be involved in both activation under low nitrate conditions and repression under high N conditions^25^. Although the NIGT1-mediated repression appeared to be more evident in the presence of high concentration of nitrate (Supplementary Fig. 5), further analyses would be necessary to clarify the role of NIGT1 in the presence of high concentrations of nitrate.

Our findings highlight a new mechanism to explain modulations of nitrate response. The presence of conserved NIGT1 sites in the promoters of *Arabidopsis* NIGT1-clade genes and Os*NIGT1* suggests that the NIGT1-mediated regulatory mechanism prevails among various plant species. In fact, the maize homologue of Os*NIGT1* (GRMZM2G016370), which was annotated as MYB-like gene, was upregulated by high N supply^52^. Hence, even if nitrate-inducible gene expression is also downregulated by other N metabolite-dependent pathways^25,26,51,53^ or degradation of the activated forms of NLPs (Supplementary Fig. 11), the NIGT1 pathway would be a useful target for modification of nitrate responses to improve N utilisation and productivity in various crop species.

Our findings also uncovered a molecular mechanism for integration of nitrate and P-starvation signalling (Fig. 9b). Plants require nutrients in appropriate ratios, because deficiency of one particular nutrient may lead to excess accumulation of other nutrients. Similarly, abundance of one nutrient may lead to deficiencies of other nutrients. Such interdependence is exhibited by N and P, both of which are required in abundance for optimal plant growth and are hence found in large quantities in plant fertilisers. P limitation inhibits N uptake in tobacco^40^, barley^38^ and maize^39^, and is accompanied by an accumulation of amino acids^40,54,55^. These phenomena suggest that plants possess regulatory mechanisms to coordinate the uptake and utilisation of nutrients to maintain metabolic homeostasis. NLA ubiquitin E3 ligase was shown to mitigate nitrate limitation-induced P accumulation^56^ through the degradation of PHT1 phosphate transporters^57^, and phenotypic analysis implicated NIGT1s in integration of N and P signals^36^. However, elucidation of N and P mechanisms coordinating N and P metabolism remained largely elusive. In the model based on our findings (Fig. 9b), under the P-sufficient condition, PHR1 interacts with SPXs, P sensor proteins, and inhibitors for PHR1^58,59^, and NIGT1*-*clade genes are not activated. Under P-starved conditions, PHR1 is released from SPXs and promotes expression of NIGT1-clade genes. Therefore, nitrate uptake is partly decreased by P-starvation via the PHR1-NIGT1-NRT2.1 pathway. The inhibition of N uptake lowers the rate of anabolism and thus decreases the demand for P. Hence, the regulation of N uptake by the PHR1-NIGT1 pathway may be a strategy to adapt to P deficiency, although other mechanisms, such as general reductions in biosynthesis, likely contribute to reductions in N uptake under P-deficient conditions.

NIGT1*-*clade gene expression was more strongly induced by nitrate signalling than by P-starvation signalling (Figs. 2, 7). Thus, NIGT1s primarily act as modulators for nitrate response, and the role in P-starvation signalling is presumably ancillary. This is consistent with fundamental regulation of nitrate uptake by N-nutrient condition and secondary regulation by P availability. Unexplained observations, such as the reduction of *NRT2*.*1* promoter activity in the absence of NIGT1-binding, indicate that the model shown in Fig. 9b requires refinement by additional analyses and incorporation of other factors. However, the key finding of the current study is that two independent transcriptional cascades for nitrate and P-starvation signalling are integrated via expression control of NIGT1-clade genes.

Phenotypic analysis revealed that constitutive overexpression of *NIGT1*.*1* severely affected growth, leading to smaller shoots and shorter primary roots, irrespective of the nitrate concentration (Supplementary Fig. 12a, b). Conversely, quadruple *nigt1* mutants displayed slight reductions in total lateral root length, although shoot fresh weight and primary root length were similar to those of the wild type (Supplementary Fig. 12). Previous phenotypic analysis indicated that *NIGT1*.*3* and *NIGT1*.*4* were involved in primary root shortening under P-deficient conditions^35,36^ and *NIGT1*.*2* was involved in the promotion of lateral root growth^37^. These observed phenotypes might suggest differences in expression patterns and/or target genes of different NIGT1s. Although *NLP7* and NIGT1-clade genes appear to be expressed in most cell types in roots, the cells where *NIGT1*.*1* and *NIGT1*.*2* are predominantly expressed appear to be are somewhat different from the cells where *NIGT1*.*3* and *NIGT1*.*4* expression are predominant (Supplementary Fig. 6b). Detailed analyses including cell-type specificity of the expression of NIGT1-clade genes and NIGT1 protein stability^36^ would allow our model to be refined to explain the observed phenotypes.

## Methods

### Plant materials

*Arabidopsis* ecotype Columbia (Col) was used as the wild type. T-DNA insertion lines GABI_267G03^60^ (*nigt1*.*1-1*), SALK_044835C (*nigt1*.*2-1*/*hho2-1*)^37^, SALK_070096 (*nigt1*.*2-2*/*hho2-2*)^37^, SAIL_28_D03 (*nigt1*.*3*/*hho1-1*)^36^, WiscDsLoxHS231_10C (*nigt1*._*3*_^*−*^*2*), SALK_067074 (*nigt1*.*4-1/hrs1-1*)^35^, SALK_067629C (*phr1*)^37^, SALK_036557 (*nlp6*)^61^, SALK_026134 (*nlp7-1*)^11^ and SAIL_731_B09 (*phl1*)^42^ were obtained from the *Arabidopsis* Biological Resource Center or Max-Planck Institute (Supplementary Fig. 8). The *nigt1* quadruple, *nlp6 nlp7-1* double and *phr1 phl1* double mutants were generated by crossing T-DNA lines and selecting plants homozygous for all T-DNA alleles using PCR-based genotyping. Primers used for genotyping are listed in Supplementary Table 2. The transgenic lines expressing the chimeric repressor form of NLP6 (NLP6-SUPRD) were described previously^8^. The NIGT1.2-GFP-OX line that was generated by transformation of Col plants^46^ expresses NIGT1.2-GFP fusion protein under the control of the 35S promoter.

### Plant growth conditions

Seeds were stratified at 4 °C for 3−4 days. Plants were grown at 23 °C with continuous illumination (60 µmol m^−2^ s^−1^) under a variety of nutrient conditions denoted for each experiment. Dark growth treatments are noted in the relevant experimental procedures. Agar media comprised 0.5× Murashige and Skoog salts^62^ (1/2MS) buffered with 0.5 g l^−1^ MES-KOH (pH 5.7) and solidified with 0.8% agar. Liquid media comprised 0.1× MS salts (1/10MS) buffered with 0.1 g l^−1^ MES-KOH (pH 5.7). Supplementation with sucrose or K^+^ (as K_2_SO_4_) is indicated where relevant. When modification of N source or concentration was required, KNO_3_ and NH_4_NO_3_ in MS salts were omitted or replaced with the indicated N source. For P-starvation treatment, KH_2_PO_4_ was omitted and the concentration of FeSO_4_ was lowered to 0.4 µM to avoid possible detrimental effects of excess iron^63^.

### Plasmid construction

Reporter plasmids for transient assays, pNRT2.1p-LUC and pNIGT1pro-LUC, harboured the *NRT2*.*1* or *NIGT1* gene promoter upstream of the *LUC* gene. To construct plasmids, a 1.3 kb *NRT2*.*1* promoter fragment and 1 kb NIGT1 promoter fragments were amplified from genomic DNA by PCR and cloned between the *Hin*dIII and *Nco*I sites of pJD301^64^. *NRT2*.*1* and *NIGT1*.*1* promoter fragments carrying mutations were produced by the overlapping PCR method and similarly cloned into pJD301 to generate (mut1)-LUC, (mut2)-LUC, (mut3)-LUC, (mut1+2)-LUC, (mut1+2+3)-LUC, (D1mut)-LUC, (P1mut)-LUC, (P2mut)-LUC, (P1P2mut)-LUC, and (D1P1P2mut)-LUC plasmids and pNIGT1pro-LUC-related plasmids. The effector plasmids for the transient expression of MYC-tagged LBD37, NIGT1.1-1.4, and PHR1 in protoplasts were generated by replacing the cDNA of *EIN3* in p35S-C4PPDK-EIN_3_^−^MYC^65^ with *LBD37*, *NIGT1*.*1-1*.*4*, or *PHR1* cDNA obtained by RT-PCR. The NLP7 expression plasmid and the internal control plasmid pUBQ10-GUS were described previously^8^. The pET32-NIGT1.1(DBD) plasmid for production of recombinant NIGT1.1 protein in *E*. *coli* was generated by insertion of a DNA fragment encoding the GARP domain of NIGT1.1 between the *Nco*I and *Xho*I sites of pET32. The pET32-based plasmid for the production of recombinant NLP7(DBD) was described previously^8^. A binary plasmid for overexpression of *NIGT1*.*1* tagged with six copies of the MYC epitope-encoding sequence was generated by replacing the 35SΩ promoter sequence and *NLP6* cDNA in pCB302HYG-35SΩ-NLP6-MYC^10^ with the 1.5 kb sequence of the *UBQ10* promoter and *NIGT1*.*1* cDNA. A binary plasmid for expression of PHR1 tagged with six copies of the MYC epitope-tag was generated by replacing NLP6 cDNA in pCB302HYG-35SΩ-NLP6-MYC^10^ with PHR1 cDNA. Binary plasmids for the expression of *LUC* under the control of wild-type or mutated *NRT2*.*1* promoters were generated by replacing the 35SΩ sequence of pCB302-35SΩ-LUC^66^ with the *NRT2*.*1* promoter fragments of *NRT2*.*1*p-LUC and (mut1+2)-LUC plasmids. All PCR products were verified by DNA sequencing. Primers are listed in Supplementary Table 2.

### Co-transfection assay

*Arabidopsis* Col seeds on a urethane sponge wetted with 1/10MS solution were incubated under continuous illumination for 1 day and subsequently in the dark for 2 days. Etiolated seedlings were then hydroponically grown with 1/10MS solution in the light for 17 days and then with N-free 1/10MS solution supplemented with 0.5 mM K_2_SO_4_ for an additional 3 days for N-starvation treatment. Preparation of mesophyll protoplasts and co-transfection of plasmids into protoplasts were performed as described in Yoo et al.^67^. Co-transfections with 2×10^4^ protoplasts were carried out with 20 µg total of plasmids (6 µg of the reporter plasmid, 12 µg of the effector plasmid, and 2 µg of pUBQ10-GUS). For simultaneous introduction of two effector plasmids at different ratios, 2.6 µg of the reporter plasmid, 5.2 µg (for ratio: 1) or 10.4 µg (for ratio: 2) of the effector plasmids, and 2 µg of pUBQ10-GUS was used. Total plasmid amounts for each transfection were adjusted to 20 µg with the empty (none) plasmid. Transfected protoplasts were incubated in WI solution^67^ in the dark overnight. For nitrate treatment, transfection of N-starved protoplasts was similarly performed but with 4×10^4^ protoplasts and 40 µg of plasmids. One half each of the transfected protoplasts was incubated in WI solution supplemented with KCl or KNO_3_ in the dark overnight. The final concentration of KCl and KNO_3_ in WI solution was noted in the relevant figure legends. LUC and β-glucuronidase (GUS) assays were performed with the Luciferase Assay System (Promega) and 4-methylumbelliferyl-β-d-glucuronide, respectively. LUC activities were normalised to GUS activities produced by the reference plasmid pUBQ10-GUS. Triplicate biological samples were analysed with consistent results. Co-transfection assays were performed at least two times with similar results.

### EMSA

DNA probes were prepared using two rounds of PCR. The first round of PCR was performed with NRT2.1p-LUC, (mut1)-LUC, (mut2)-LUC or (mut3)-LUC plasmids as template and specific primer pairs. Reverse primers for NIGT1-binding assays also carried the M13 reverse primer sequence, and the primers for NLP7-binding assays contained OligoY or Z sequence (Supplementary Table 2). After purification of PCR products through electrophoresis with an acrylamide gel, the second round of PCR was performed with the purified PCR product as a template, using the same forward primer and a biotin-labelled M13 primer (for NIGT1-binding assays) or biotin-labelled OligoY and OligoZ (for NLP7-binding assays). The secondary PCR products were purified using the Wizard^®^ SV Gel and PCR Clean-Up System (Promega) and used as biotin-labelled probes. The OsNIGT1 probe used as a positive control was similarly produced by two rounds of PCR. This probe corresponded to a region of the Os*NIGT1* promoter (−251 to −95 relative to the translation start codon) and contained one conserved and one nonconserved NIGT1-binding sequence^17^. Recombinant Trx- and His-tagged NIGT1.1(DBD) and NLP7(DBD) proteins were produced in *E. coli* BL21(DE3) cells transformed with the pET32-NIGT1.1(DBD) and pET32-NLP7(DBD) plasmids, respectively, and purified using Complete His-tag Purification Resin (Roche). Binding of the recombinant protein (25–50 ng) to DNA probes (2.5–5 ng) was carried out in a reaction mixture containing 0.5 µg of poly[d(I-C)], 20 mM HEPES-KOH (pH 7.6), 3 mM MgCl_2_, 30 mM KCl, 20 mM NaCl, 2 mM dithiothreitol, 0.5 mM EDTA (pH 8.0), 10% glycerol, and 0.1× EDTA-free Complete (Roche). Electrophoresis was performed with native 5% polyacrylamide gels in 0.5× TBE. Probe DNAs transferred onto the positively charged nylon membranes (Biodyne B, PALL) were detected with streptavidin-horseradish peroxidase (Pierce). The results were verified by two independent experiments (Pierce).

### RT-qPCR analysis

RT-qPCR analysis of nitrate induction was performed with *Arabidopsis* seedlings that were grown in N-free 1/10MS solution supplemented with 0.5 mM ammonium succinate as a N source and 0.5% sucrose for 5 days. KNO_3_ or other N compounds were supplied to induce related gene expression, and seedlings were collected after 1, 3 or 6 h. For RT-qPCR analysis with P-starved plants, Col and *phr1 phl1* seeds were germinated on a urethane sponge wetted with N-free 1/10MS medium supplemented with 0.2 mM KNO_3_, and seedlings were grown under illumination for 1 day and subsequently in the dark for 2 days. Etiolated seedlings were then hydroponically grown with the same medium for 13 days. Plants were then transferred to the same fresh medium or fresh medium without P and grown for 5 days. Total RNA from seedlings was prepared with ISOGEN reagent (NIPPONGENE, Tokyo, Japan) or with an ISOSPIN plant RNA kit (NIPPONGENE). RT reactions were performed using SuperScriptII reverse transcriptase (Thermo Fisher Scientific), and qPCR was performed with a StepOne Plus™ Real Time PCR System (Applied Biosystems) and the KAPA SYBR Fast qPCR Kit (KAPA Biosystems). Triplicate biological samples were analysed with consistent results, and the results were verified by two independent experiments.

### Generation of transgenic plants

Transformation of *Arabidopsis* was performed by the floral dip method using Agrobacterium strain GV3101 (pMP90)^68^. Lines that exhibited a segregation of 3:1 for glufosinate ammonium or hygromycin B resistance at the T_2_ generation were selected, and T3 progenies of the lines that were homozygous for the introduced gene were used for all analyses in this study.

### ChIP assay

For ChIP analysis with NIGT1.1-OX #9 plants, seedlings were grown for 10 days on agar plates that contained 1/2MS salts, 3 mM MES-KOH (pH 5.8), 0.5% sucrose and 0.8% agar. For ChIP analysis with PHR1-OX plants, etiolated seedlings were generated by cultivation for 1 day under continuous illumination and then 2 days in the dark. Plants were then hydroponically grown with 1/10MS solution for 13 days and then 1/10MS solution without P for 5 days. ChIP was performed using the protocol of Saleh et al.^69^ with slight modifications. In brief, after cross-linking proteins to DNA and isolation and lysis of nuclei, DNA sonication was performed with a BIORUPTOR^®^II (COSMO BIO CO., LTD, Tokyo, Japan) at high mode, 10× 30 s, with 30-s intervals. Immunoprecipitation was carried out with Protein A or G-agarose beads (Roche) conjugated to 5 µg of anti-MYC antibody (clone 9E10, Millipore #05-419) overnight at 4 °C. DNA recovered from agarose beads was purified using a DNeasy Plant Mini Kit (Qiagen). qPCR was performed with a StepOne Plus™ Real Time PCR System (Applied Biosystems) and the KAPA SYBR Fast qPCR Kit (KAPA Biosystems). Three or four biological samples were analysed with consistent results.

### Imaging of LUC activity in vivo

For in vivo LUC imaging of NH_4_NO_3_-treated plants harbouring the NRT2.1p-LUC and (mut1+2)-LUC construct, we introduced these constructs into the wild-type *Arabidopsis* (Col). Five T3 seedlings of each resultant transgenic line were grown in 3 ml of N-free 1/10MS medium supplemented with 2 mM KNO_3_ and 0.5% sucrose in one well of six-well plates for 5 days. Ammonium nitrate solution was then added into the medium at a final concentration of 10 mM, and seedlings were incubated for 24 h. An equal amount of water was added to control seedlings. For analysing effects of different concentrations of nitrate, three seedlings were grown in 5 ml of N-free 1/10MS medium supplemented with indicated concentrations of KNO_3_ and 0.5% sucrose in one well of six-well plates for 6 days. For in vivo LUC imaging of P-starved *NRT2*.*1*p-LUC and (mut1+2)-LUC plants, seedlings were grown in N-free 1/10MS medium supplemented with 1 mM KNO_3_ and 0.5% sucrose for 5 days. The medium was then changed to N-free 1/10MS medium supplemented with 0.5 mM KNO_3_, 0.25 mM K_2_SO_4_ and 0.5% sucrose, and seedlings were incubated for 1 day. Seedlings were then grown with 1/10MS-based medium with or without P. Nutrient solutions were then changed daily. For in vivo imaging of LUC activity, luciferin potassium salt (Wako, Osaka, Japan) was added to the medium at a final concentration of 1 mM. After incubating in the dark for 10 min, luminescence from the seedlings was detected using an Amersham Imager 600 (GE Healthcare) with 60 min exposure. The obtained 16 bit images were converted to pseudo-colour using ImageJ software (NIH, USA).

### Genome-wide survey of genes under the control of NIGT1s

*Arabidopsis* genes regulated by nitrate were identified using published data^7^. Nitrate-upregulated genes were those that met both of the following criteria: (1) the expression level in KNO_3_-treated samples was more than twofold higher than expression in control (no treatment) samples; and (2) the expression level in KNO_3_-treated samples was more than twofold higher than expression in KCl-treated samples. Nitrate-downregulated genes were those that met both of the following criteria: (1) the expression level was more than twofold lower in KNO_3_-treated samples than in control (no treatment) samples; and (2) the expression level in KNO_3_-treated samples was more than twofold lower than expression in KCl-treated samples. Genes downregulated by NIGT1.2-OX were described in Kiba et al.^46^ (accession number GSE100903 in Gene Expression Omnibus). Venn diagrams with numbers of selected genes were generated by BioVenn.

### Quantification of cytokinin

Seedlings were grown in N-free 1/2MS liquid medium supplemented with 1% sucrose at 22 ℃ under continuous light with rotation (120 rpm) for 4 days and treated with 1 mM KCl or 1 mM KNO_3_ for 12 h before harvest. Extraction and determination of cytokinin was performed with the same system constructed in Kojima et al.^70^ In brief, the extract obtained from about 100 mg of seedlings with the extraction solvent (methanol:formic acid:water = 15:1:4) was mixed with internal standards labelled with stable isotopes and loaded onto an Oasis MCX 96-Well Plate 30 mg (Waters) equilibrated with 1 M formic acid. After washing with 1 M formic acid and then with methanol, cytokinin nucleotides were eluted with 0.35 M ammonia, and then cytokinin nucleobases were eluted with 0.35 M ammonia in 60% (v/v) methanol. The eluate containing cytokinin nucleotides was reconstituted with 0.84 ml of 0.1 M *N*-cyclohexyl-2-aminoethanesulfonic acid-NaOH (pH 9.8) and treated with alkaline phosphatase (17 µl of 1 U μl^−1^, Oriental Yeast Co. Ltd., Tokyo, Japan) for 1 h at 37 °C. After the treatment, 93 μl of 10× Tris-buffered saline and 46 μl of 1 M HCl were added into the reaction mixture. Then, the mixture was desalted by passing through a water-equilibrated Oasis HLB 96-Well Plate 30 mg (Waters), and cytokinin nucleotides were eluted with methanol. After evaporation of methanol, cytokinin nucleotides were dissolved in 50 μl of 0.1% acetic acid and were subjected to ultra-performance liquid chromatography coupled with a tandem quadrupole mass spectrometer equipped with an electrospray interface (UPLC-ESI-qMS/MS; AQUITY UPLC™ System/Quattro Ultima Pt, Waters) analysis with an ODS column (AQUITY UPLC BEH C 18, Waters). On the other hand, solvents of the eluate containing cytokinin nucleobases were evaporated, and cytokinin nucleobases were dissolved in 50 μl of 0.1% acetic acid and subjected to UPLC-ESI-qMS/MS analysis. Data were processed by the MassLynx™ software with QuanLynx™ (Waters).

### ^15^NO_3_^−^ uptake

Etiolated seedlings generated by growth under illumination for 1 day and in the dark for 2 days were then hydroponically grown with 1/10MS solution in the light for 13 days. Plants were then grown with 1/10MS solution with or without P for 5 days. Plant roots were submerged in 0.1 mM CaSO_4_ for 1 min and then medium containing 0.2 mM K^15^NO_3_ for 5 min. After washing in 0.1 mM CaSO_4_ for 1 min^28^, roots were collected and dried. N content and ^15^N ratio were analysed by SI Science Co. Ltd. (Tokyo, Japan). Five biological replicates were analysed with consistent results.

### Immunoblot analysis

Seedlings of the transgenic lines expressing MYC-tagged NLP7^10^ were grown in N-free 1/10MS solution supplemented with 0.5 mM ammonium succinate (Nitrogen-free 1/10 MS salts, 0.1 g l^−1^ MES-KOH (pH 5.7), 0.5% sucrose, 0.5 mM ammonium succinate) for 4 days and the solution was then changed to the same fresh solution but containing 10 mM KCl or KNO_3_. Seedlings were collected 0, 0.5, 1, 3, 6 and 24 h after the change of the solution. The seedlings were weighed, frozen in liquid nitrogen and ground using a Multibeads Shocker (Yasui Kikai, Osaka, Japan). The ground samples were suspended in 10 volume of 1× Laemmli sample buffer supplemented with EDTA-free protease inhibitor cocktail (Roche) and heated at 95 °C for 30 s. Samples were then subjected to SDS-PAGE and immunoblotting with anti-MYC (Millipore, 05-419; 1:1000) and anti-Histone H3 (Abcam, ab1791; 1:5000) antibodies.

### Phenotypic analysis

Seeds of Col, NIGT1 overexpressors and *nigt1* quadruple mutants were germinated on 1/2MS plates (1/2MS salts, 0.5 g l^−1^ MES-KOH (pH 5.7), 1% sucrose and 0.8% agar). Seedlings were grown on plates for 3 days and then transferred to vertically placed test plates (N-free 1/2MS salts, 0.5 g l^−1^ MES-KOH (pH 5.7), 1% sucrose and 0.8% agar) supplemented with the indicated concentrations of KNO_3_ and NH_4_NO_3_ and grown for 6 days. The upper 2 cm part of the agar medium was removed from test plates to avoid contact of shoots with the medium. Images of agar plates were taken using an Amersham Imager 600 (GE Healthcare), and root lengths were measured using ImageJ software (NIH, USA). The results were verified by two independent experiments.

### Data availability

The datasets generated and/or analysed during the current study are available from the corresponding author on reasonable request.

## Electronic supplementary material

Supplementary Information

Description of Additional Supplementary Files

Supplementary Data 1

Supplementary Data 2

## References

[1] Marschner, H. *Mineral Nutrition of Higher Plants* (Academic Press, London, 1995).

[2] Xu G, Fan X, Miller AJ (2012). Plant nitrogen assimilation and use efficiency. Annu. Rev. Plant Biol..

[3] Crawford NM (1995). Nitrate: nutrient and signal for plant growth. Plant Cell.

[4] Krapp A (2014). Nitrate transport and signalling in *Arabidopsis*. J. Exp. Bot..

[5] Tsay YF, Ho CH, Chen HY, Lin SH (2011). Integration of nitrogen and potassium signaling. Annu. Rev. Plant Biol..

[6] Wang R, Okamoto M, Xing X, Crawford NM (2003). Microarray analysis of the nitrate response in *Arabidopsis* roots and shoots reveals over 1,000 rapidly responding genes and new linkages to glucose, trehalose-6-phosphate, iron, and sulfate metabolism. Plant Physiol..

[7] Scheible WR (2004). Genome-wide reprogramming of primary and secondary metabolism, protein synthesis, cellular growth processes, and the regulatory infrastructure of *Arabidopsis* in response to nitrogen. Plant Physiol..

[8] Konishi M, Yanagisawa S (2013). *Arabidopsis* NIN-like transcription factors have a central role in nitrate signalling. Nat. Commun..

[9] Marchive C (2013). Nuclear retention of the transcription factor NLP7 orchestrates the early response to nitrate in plants. Nat. Commun..

[10] Liu KH (2017). Discovery of nitrate-CPK-NLP signalling in central nutrient-growth networks. Nature.

[11] Castaings L (2009). The nodule inception-like protein 7 modulates nitrate sensing and metabolism in *Arabidopsis*. Plant J..

[12] Wang R, Xing X, Wang Y, Tran A, Crawford NM (2009). A genetic screen for nitrate regulatory mutants captures the nitrate transporter gene NRT1.1. Plant Physiol..

[13] Karve R, Suárez-Román F, Iyer-Pascuzzi AS (2016). The transcription factor NIN-LIKE PROTEIN7 controls border-like cell release. Plant Physiol..

[14] Yan D (2016). NIN-like protein 8 is a master regulator of nitrate-promoted seed germination in *Arabidopsis*. Nat. Commun..

[15] Alvarez JM (2014). Systems approach identifies TGA1 and TGA4 transcription factors as important regulatory components of the nitrate response of *Arabidopsis thaliana* roots. Plant J..

[16] Sawaki N (2013). A nitrate-inducible GARP family gene encodes an auto-repressible transcriptional repressor in rice. Plant Cell Physiol..

[17] Rubin G, Tohge T, Matsuda F, Saito K, Scheible WR (2009). Members of the LBD family of transcription factors repress anthocyanin synthesis and affect additional nitrogen responses in *Arabidopsis*. Plant Cell.

[18] Krouk G, Mirowski P, LeCun Y, Shasha DE, Coruzzi GM (2010). Predictive network modeling of the high-resolution dynamic plant transcriptome in response to nitrate. Genome Biol..

[19] Maeda S, Konishi M, Yanagisawa S, Omata T (2014). Nitrite transport activity of a novel HPP family protein conserved in cyanobacteria and chloroplasts. Plant Cell Physiol..

[20] Sato T (2017). Direct transcriptional activation of *BT* genes by NLP transcription factors is a key component of the nitrate response in *Arabidopsis*. Biochem. Biophys. Res. Commun..

[21] Konishi M, Yanagisawa S (2014). Emergence of a new step towards understanding the molecular mechanisms underlying nitrate-regulated gene expression. J. Exp. Bot..

[22] Li W (2007). Dissection of the *AtNRT2*.*1:AtNRT2*.*2* inducible high-affinity nitrate transporter gene cluster. Plant Physiol..

[23] Orsel M, Eulenburg K, Krapp A, Daniel-Vedele F (2004). Disruption of the nitrate transporter genes *AtNRT2*.*1* and *AtNRT2*.*2* restricts growth at low external nitrate concentration. Planta.

[24] Zhuo D, Okamoto M, Vidmar JJ, Glass AD (1999). Regulation of a putative high-affinity nitrate transporter (Nrt2;1At) in roots of *Arabidopsis thaliana*. Plant J..

[25] Girin T (2007). Identification of a 150 bp cis-acting element of the *AtNRT2*.*1* promoter involved in the regulation of gene expression by the N and C status of the plant. Plant Cell Environ..

[26] Nazoa P (2003). Regulation of the nitrate transporter gene *AtNRT2*.*1* in *Arabidopsis thaliana*: responses to nitrate, amino acids and developmental stage. Plant Mol. Biol..

[27] Vidmar JJ, Zhuo D, Siddiqi MY, Glass AD (2000). Isolation and characterization of *HvNRT2*.*3* and *HvNRT2*.*4*, cDNAs encoding high-affinity nitrate transporters from roots of barley. Plant Physiol..

[28] Krapp A (1998). Expression studies of *Nrt2:1Np*, a putative high-affinity nitrate transporter: evidence for its role in nitrate uptake. Plant J..

[29] Guan P (2014). Nitrate foraging by *Arabidopsis* roots is mediated by the transcription factor TCP20 through the systemic signaling pathway. Proc. Natl. Acad. Sci. USA.

[30] Widiez T (2011). HIGH NITROGEN INSENSITIVE 9 (HNI9)-mediated systemic repression of root NO_3_^−^ uptake is associated with changes in histone methylation. Proc. Natl. Acad. Sci. USA.

[31] Krouk G, Tillard P, Gojon A (2006). Regulation of the high-affinity NO_3_^−^ uptake system by NRT1.1-mediated NO_3_^−^ demand signaling in *Arabidopsis*. Plant Physiol..

[32] Bi YM, Wang RL, Zhu T, Rothstein SJ (2007). Global transcription profiling reveals differential responses to chronic nitrogen stress and putative nitrogen regulatory components in *Arabidopsis*. Bmc Genom..

[33] Kramer V, Lahners K, Back E, Privalle LS, Rothstein S (1989). Transient accumulation of nitrite reductase mRNA in maize following the addition of nitrate. Plant Physiol..

[34] Yanagisawa S (2013). Characterization of a nitrate-inducible transcriptional repressor NIGT1 provides new insights into DNA recognition by the GARP family proteins. Plant Signal. Behav..

[35] Liu H (2009). Overexpressing *HRS1* confers hypersensitivity to low phosphate-elicited inhibition of primary root growth in *Arabidopsis thaliana*. J. Integr. Plant Biol..

[36] Medici A (2015). AtNIGT1/HRS1 integrates nitrate and phosphate signals at the *Arabidopsis* root tip. Nat. Commun..

[37] Nagarajan VK, Satheesh V, Poling MD, Raghothama KG, Jain A (2016). *Arabidopsis* MYB-related HHO2 exerts a regulatory influence on a subset of root traits and genes governing phosphate homeostasis. Plant Cell Physiol..

[38] Lee RB (1982). Selectivity and kinetics of ion uptake by barley plants following nutrient deficiency. Ann. Bot..

[39] de Magalhaes JV (1998). Nitrate uptake by corn under increasing periods of phosphorus starvation. J. Plant Nutr..

[40] Rufty TW, Mackown CT, Israel DW (1990). Phosphorus stress effects on assimilation of nitrate. Plant Physiol..

[41] Rubio V (2001). A conserved MYB transcription factor involved in phosphate starvation signaling both in vascular plants and in unicellular algae. Genes Dev..

[42] Bustos R (2010). A central regulatory system largely controls transcriptional activation and repression responses to phosphate starvation in *Arabidopsis*. PLoS Genet..

[43] Husbands A, Bell EM, Shuai B, Smith HM, Springer PS (2007). LATERAL ORGAN BOUNDARIES defines a new family of DNA-binding transcription factors and can interact with specific bHLH proteins. Nucleic Acids Res..

[44] Okamoto M, Vidmar JJ, Glass AD (2003). Regulation of *NRT1* and *NRT2* gene families of *Arabidopsis thaliana*: responses to nitrate provision. Plant Cell Physiol..

[45] Wirth J (2007). Regulation of root nitrate uptake at the NRT2.1 protein level in *Arabidopsis thaliana*. J. Biol. Chem..

[46] Kiba, T. et al. Repression of nitrogen-starvation responses by *Arabidopsis* GARP-type HRS1 sub/HRS1 subfamily members. *Plant Cell* 10.1105/tpc.17.00810 (2018). 10.1105/tpc.17.00810 PMC5969275 29622567

[47] Kiba T, Takei K, Kojima M, Sakakibara H (2013). Side-chain modification of cytokinins controls shoot growth in *Arabidopsis*. Dev. Cell.

[48] Orsel M (2006). Characterization of a two-component high-affinity nitrate uptake system in *Arabidopsis*. Physiology and protein−protein interaction. Plant Physiol..

[49] Kiba T, Kudo T, Kojima M, Sakakibara H (2011). Hormonal control of nitrogen acquisition: roles of auxin, abscisic acid, and cytokinin. J. Exp. Bot..

[50] Gniazdowska A, Mikulska M, Rychter AM (1998). Growth, nitrate uptake and respiration rate in bean roots under phosphate deficiency. Biol. Plant..

[51] Vincentz M, Moureaux T, Leydecker MT, Vaucheret H, Caboche M (1993). Regulation of nitrate and nitrite reductase expression in *Nicotiana plumbaginifolia* leaves by nitrogen and carbon metabolites. Plant J..

[52] Schlüter U (2012). Maize source leaf adaptation to nitrogen deficiency effects not only N and C metabolism but also control of P homeostasis. Plant Physiol..

[53] Sonoda Y, Ikeda A, Saiki S, Yamaya T, Yamaguchi J (2003). Feedback regulation of the ammonium transporter gene family *AMT1* by glutamine in rice. Plant Cell Physiol..

[54] Schlüter U (2013). Adaptation of maize source leaf metabolism to stress related disturbances in carbon, nitrogen and phosphorus balance. BMC Genom..

[55] Huang CY (2008). Metabolite profiling reveals distinct changes in carbon and nitrogen metabolism in phosphate-deficient barley plants (*Hordeum vulgare* L.). Plant Cell Physiol..

[56] Kant S, Peng M, Rothstein SJ (2011). Genetic regulation by NLA and microRNA827 for maintaining nitrate-dependent phosphate homeostasis in *Arabidopsis*. PLoS Genet..

[57] Lin WY, Huang TK, Chiou TJ (2013). NITROGEN LIMITATION ADAPTATION, a target of microRNA827, mediates degradation of plasma membrane-localized phosphate transporters to maintain phosphate homeostasis in *Arabidopsis*. Plant Cell.

[58] Puga MI (2014). SPX1 is a phosphate-dependent inhibitor of PHOSPHATE STARVATION RESPONSE 1 in *Arabidopsis*. Proc. Natl. Acad. Sci. USA.

[59] Wild R (2016). Control of eukaryotic phosphate homeostasis by inositol polyphosphate sensor domains. Science.

[60] Rosso MG (2003). An *Arabidopsis thaliana* T-DNA mutagenized population (GABI-Kat) for flanking sequence tag-based reverse genetics. Plant Mol. Biol..

[61] Guan P (2017). Interacting TCP and NLP transcription factors control plant responses to nitrate availability. Proc. Natl. Acad. Sci. USA.

[62] Murashige T, Skoog F (1962). A revised medium for rapid growth and bio assays with tobacco tissue cultures. Physiol. Plant..

[63] Thibaud MC (2010). Dissection of local and systemic transcriptional responses to phosphate starvation in *Arabidopsis*. Plant J..

[64] Luehrsen KR, de Wet JR, Walbot V (1992). Transient expression analysis in plants using firefly luciferase reporter gene. Methods Enzymol..

[65] Yanagisawa S, Yoo SD, Sheen J (2003). Differential regulation of EIN3 stability by glucose and ethylene signalling in plants. Nature.

[66] Konishi M, Yanagisawa S (2007). Sequential activation of two Dof transcription factor gene promoters during vascular development in *Arabidopsis thaliana*. Plant Physiol. Biochem..

[67] Yoo SD, Cho YH, Sheen J (2007). *Arabidopsis* mesophyll protoplasts: a versatile cell system for transient gene expression analysis. Nat. Protoc..

[68] Clough SJ, Bent AF (1998). Floral dip: a simplified method for *Agrobacterium*-mediated transformation of *Arabidopsis thaliana*. Plant J..

[69] Saleh A, Alvarez-Venegas R, Avramova Z (2008). An efficient chromatin immunoprecipitation (ChIP) protocol for studying histone modifications in *Arabidopsis* plants. Nat. Protoc..

[70] Kojima M (2009). Highly sensitive and high-throughput analysis of plant hormones using ms-probe modification and liquid chromatographytandem mass spectrometry: An application for hormone profiling in *Oryza sativa*. Plant Cell Physiol..

