# Supplementary material for: A NIGT1-centred transcriptional cascade regulates nitrate signalling and incorporates phosphorus starvation signals in Arabidopsis
Source: Nat Commun. 2018 Apr 10;9:1376. doi: 10.1038/s41467-018-03832-6 (PMC5893545; doi:10.1038/s41467-018-03832-6)
Supplement: Supplementary file 1 — Supplementary Information [file 41467_2018_3832_MOESM1_ESM.pdf]

Supplementary Information

A NIGT1-centered transcriptional cascade regulates nitrate signalling and incorporates phosphorus starvation signals in Arabidopsis

Maeda et al.

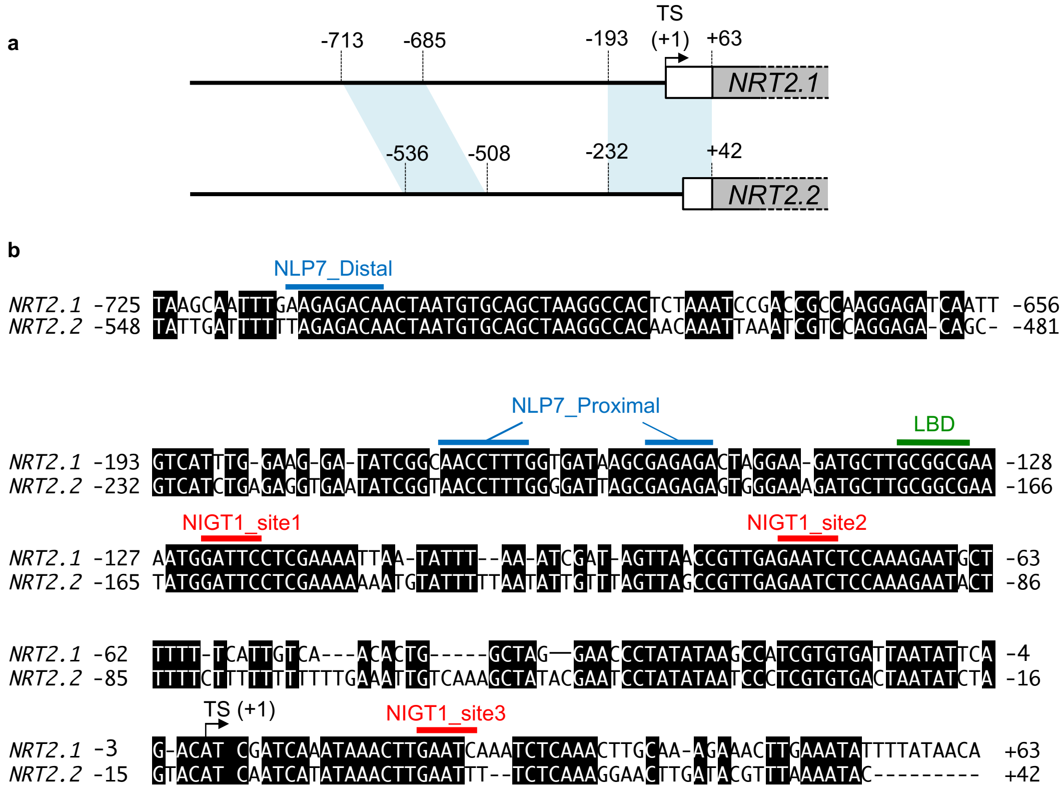

Supplementary Figure 1 | Alignment of the *NRT2.1* and *NRT2.2* promoters. Positions (**a**) and sequences (**b**) of two regions that are homologous between the *NRT2.1* and *NRT2.2* promoter sequences. In **b**, blue, red and green bars above the *NRT2.1* promoter sequence indicate sequences similar to the consensus sequences for NLP-, NIGT1-, and LBD-binding, respectively. The distal NLP-binding site (NLP7_Distal) is only similar to a part of the NLP-binding sequence in the *NIR1* promoter[1]. These sequences are conserved in the *NRT2.1* and *NRT2.2* promoters. Numbers indicate nucleotide positions relative to the transcription start site (TS) of *NRT2.1*. The 5'-untranslated regions of *NRT2.1* cDNAs registered in the database (accession numbers: Z97058, AF093754 and AF019748) are 63, 49 bp and 44 bp in length. The position of the 63th nucleotide upstream of the translational start codon is adopted as a putative TS, and numbers are relative to the putative TS.

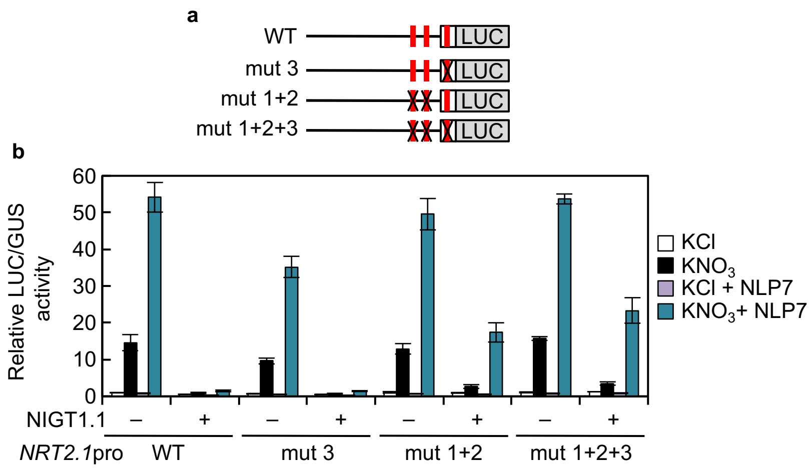

Supplementary Figure 2 | Effects of mutations within the putative NIGT1-binding site. (**a**) Reporter constructs. X indicates disrupted NIGT1 sites. (**b**) LUC activity from protoplasts that were co-transfected with the *LUC* gene fused to the wild-type or mutant *NRT2.1* promoter and the NLP7 expression vector or an empty vector and incubated in the presence of 1 mM KCl or KNO_3. LUC activity was normalised with GUS activity from the reference UBQ10-GUS plasmid and data are means ± s.d. of three biological replicates.

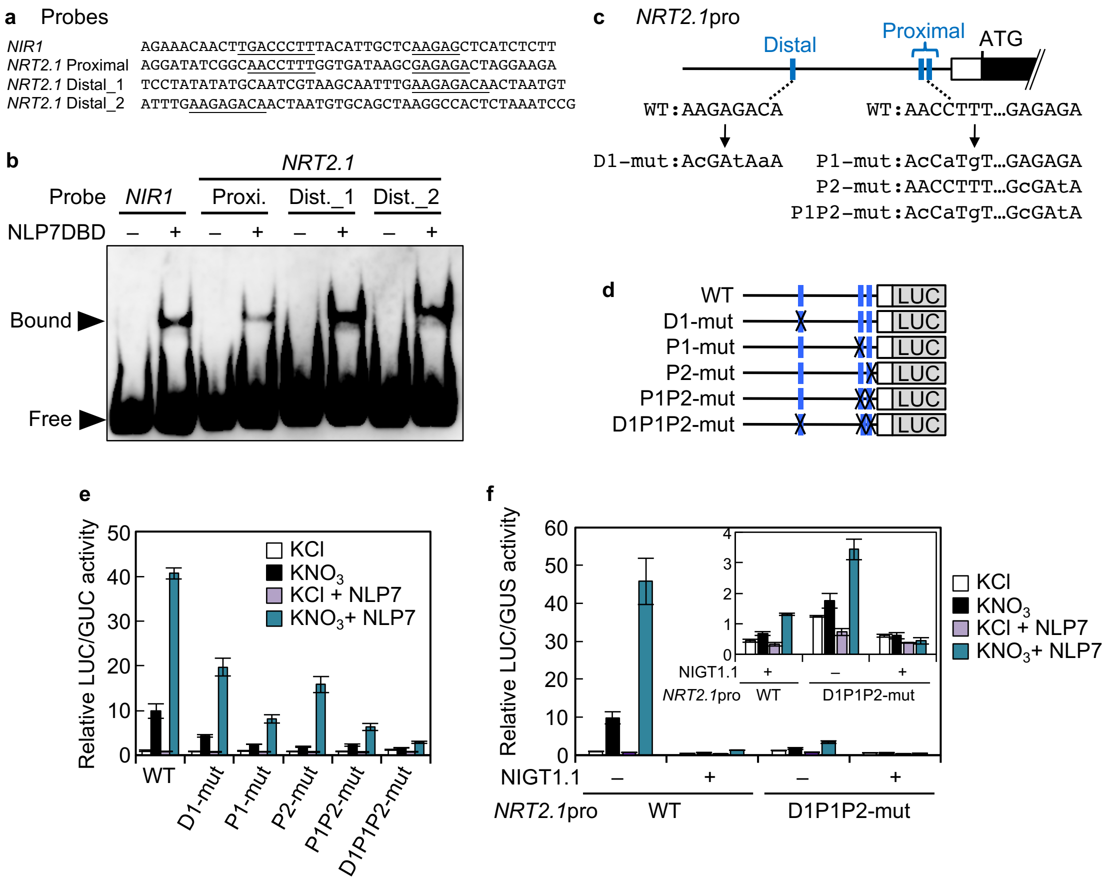

Supplementary Figure 3 | Identification of NLP-binding sites in the *NRT2.1* promoter. (**a**) DNA probes used in EMSA. (**b**) EMSA with the DNA fragments from the *NRT2.1* promoter and the DNA-binding domain of NLP7 (NLP7DBD). Distal_1 and Distal_2 probes feature the same distal NLP-binding sequence (see Supplementary Figure 1b). The NLP-binding sequence from the *NIR1* promoter[1] was used as a positive control. (**c**) A schematic representation of the 1.3 kb *NRT2.1* promoter. A horizontal line and a white box indicate the 5' upstream and the 5' untranslated regions, respectively. Blue boxes indicate two putative NLP-binding sites. Mutations in the mutated *NRT2.1* promoters are shown below the wild-type (WT) sequences. (**d**) Reporter constructs harbouring mutations on the NLP-binding sites. Mutated sites are indicated with X. (**e**,**f**) Protoplast transient assays for investigation of nitrate- and NLP7-dependent activation (**e**) and NIGT1.1-dependent repression (**f**) of mutated *NRT2.1* promoters. Protoplasts co-transfected with a reporter construct and the NLP7 or NIGT1.1 expression vector or an empty vector were incubated in the presence of 1 mM KCl or KNO_3. In **e**,**f**, LUC activity was normalised with GUS activity from the reference UBQ10-GUS plasmid and data are means ± s.d. of three biological replicates.

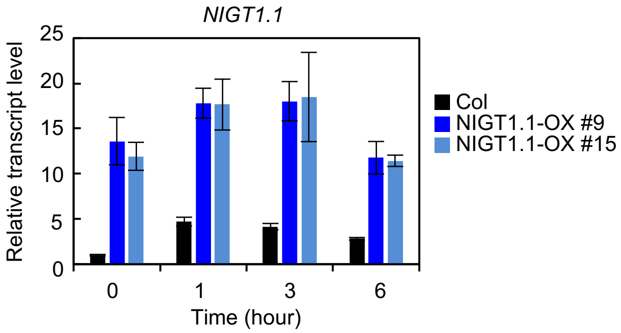

Supplementary Figure 4 | Levels of *NIGT1.1* transcripts in transgenic Arabidopsis lines harbouring the *NIGT1.1* gene under the control of the *UBQ10* promoter. Seedlings of Arabidopsis Col and two independent transgenic lines were grown with ammonium medium for 5 days, treated with KNO_3 (final concentration, 10 mM) to induce endogenous *NIGT1.1* expression, and then collected at the indicated time points. Levels of the *NIGT1.1* transcripts were quantified by RT-qPCR. The values for the NIGT1.1-OX lines indicate total levels of transcripts from endogenous and introduced *NIGT1.1* genes, whereas the values for Col seedlings indicate solely the levels of transcripts from endogenous *NIGT1.1*. Values were normalised with those of *UBQ10*, and means ± s.d. of biological triplicates are shown.

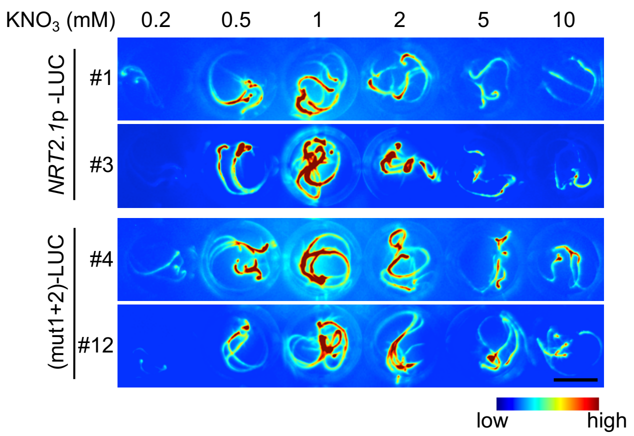

Supplementary Figure 5 | Effects of the disruption of the NIGT1-binding sites on *NRT2.1* promoter activity in the presence of different concentrations of KNO_3. Three seedlings of transgenic lines harbouring the *LUC* gene fused to the wild-type (WT) or the mutated (mut1+2) *NRT2.1* promoter were grown with indicated concentrations of KNO_3 for 6 days. Images of LUC activity *in vivo* were captured in two independent transgenic lines. Scale bar, 1 cm.

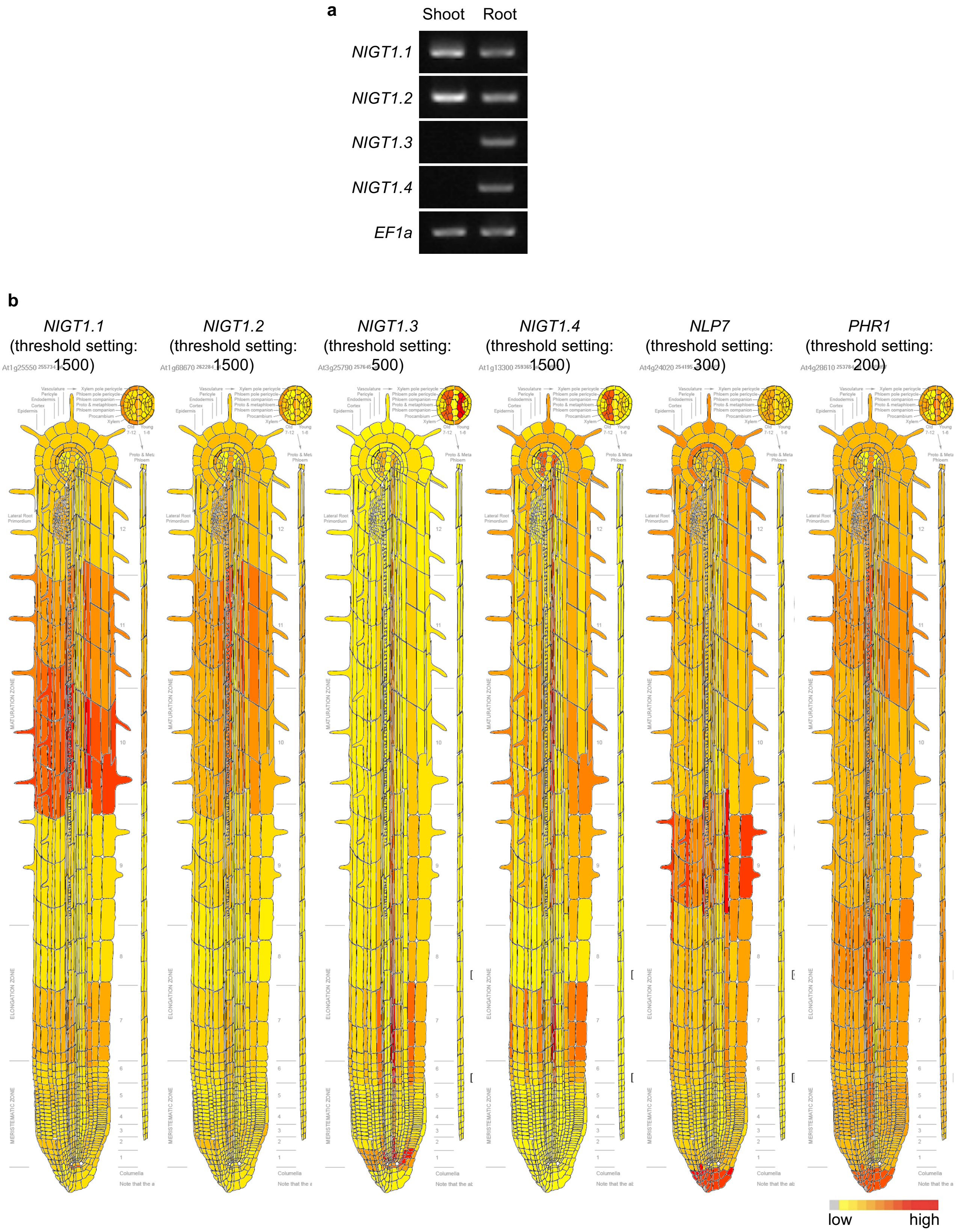

Supplementary Figure 6 | Expression of NIGT1-clade genes. (**a**) Expression in shoots and roots. RT-PCR analysis was performed with cDNA prepared from RNA isolated from shoots or roots of 14-day-old seedlings grown on 1/2MS plates supplemented with 1% sucrose. *EF1a* is a housekeeping gene and was used as a control. (**b**) Expression in roots. Images for expression of NIGT1-clade genes, *NLP7* and *PHR1* in roots, which are from Arabidopsis eFP Browser[2] (http://bbc.botany.utoronto.ca/efp/cgi-bin/efpWeb.cgi).

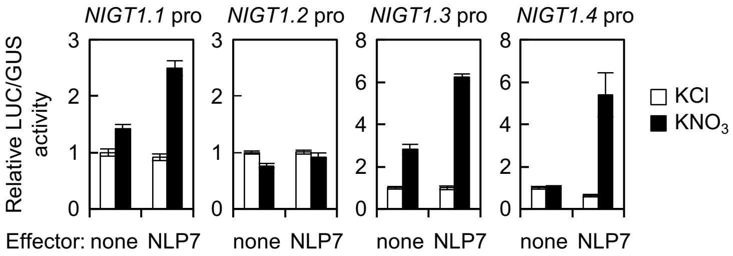

Supplementary Figure 7 | Activation of *NIGT1* gene promoters by nitrate and NLP7. Activation of promoters of *NIGT1* genes by nitrate and co-expression of NLP7 in protoplasts. The reporter plasmid containing the *LUC* gene downstream of the 1 kb promoter sequence of *NIGT1.1*, *1.2*, *1.3*, or *1.4* was co-transfected into protoplasts isolated from nitrogen-starved plants, together with the expression vector for NLP7 (NLP7) or the empty vector (none). UBQ10-GUS plasmid was included in all transfection reactions for the normalisation of transfection efficiency. Transfected protoplasts were incubated overnight with 10 mM KCl or KNO_3. LUC activity was normalised with GUS activity, and data are means ± s.d. (*n*=3).

a

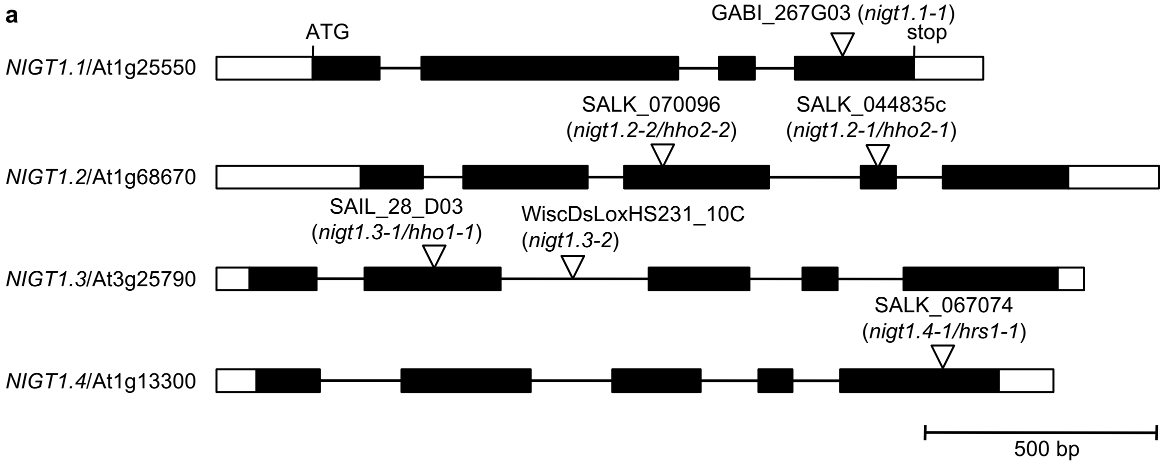

b

	NIGT1.1	*NIGT1.2*	*NIGT1.3*	*NIGT1.4*
nigtQ-1	GABI_267G03	SALK_044835c	SAIL_28_D03	SALK_067074
nigtQ-2	GABI_267G03	SALK_070096	SAIL_28_D03	SALK_067074
nigtQ-3	GABI_267G03	SALK_044835c	WiscDsLoxHS231_10C	SALK_067074
nigt123	GABI_267G03	SALK_044835c	WiscDsLoxHS231_10C	-
nigt124	GABI_267G03	SALK_070096	-	SALK_067074
nigt134	GABI_267G03	-	WiscDsLoxHS231_10C	SALK_067074
nigt234	-	SALK_070096	WiscDsLoxHS231_10C	SALK_067074

c

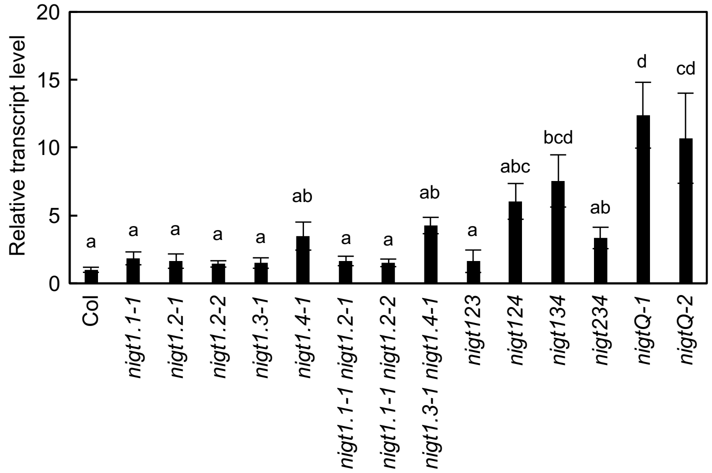

Supplementary Figure 8 | *nigt1* T-DNA lines. (**a**) Positions of T-DNA insertions in *NIGT1* genes. Boxes and horizontal lines indicate exons and introns, respectively. White and black boxes indicate untranslated and coding regions, respectively. (**b**) Combination of T-DNA insertion alleles in the *nigt1* quadruple mutants *Q-1*, *Q-2 and Q-3,* and triple mutants (*nigt123, 124, 134* and *234*). (**c**) *NRT2.1* transcript levels in single, double, triple and quadruple *nigt1* mutants. Plants were grown on 1/2MS plates supplemented with 1% sucrose for 9 days. Values are mean ± s.d. (*n*=3). Bars not sharing the same letter designation are significantly different from each other (Tukey's HSD, $p < 0.05$).

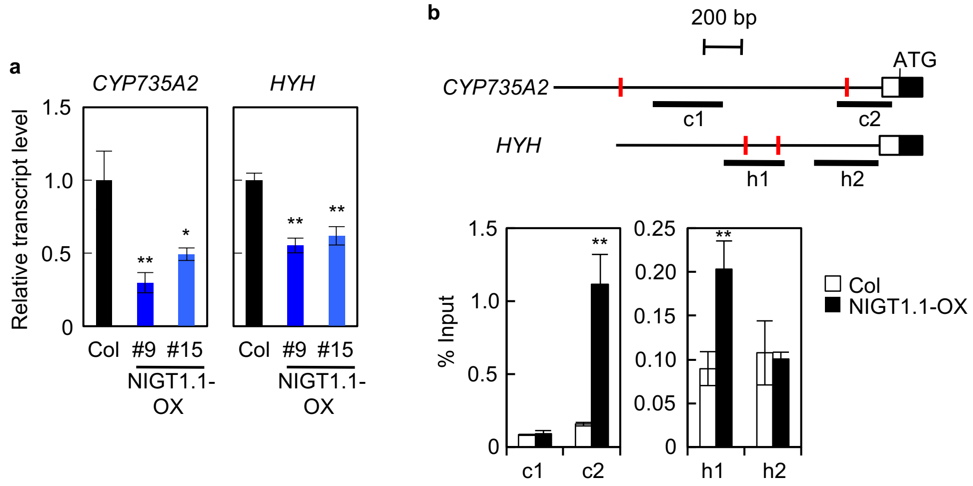

Supplementary Figure 9 | Targets of NIGT1 proteins include *CYP735A2* and *HYH*. (**a**) Reduced expression of *CYP735A2* and *HYH* in the NIGT1.1 overexpressor. RNA from seedlings of Arabidopsis Col and NIGT1.1-OX lines (#9 and #15) was used for RT-qPCR. Data are means of three biological samples ± s.d. * p <0.05, ** p <0.01 by two-tailed t-test compared with corresponding control values obtained with Col seedlings. (**b**) Binding of NIGT1.1 to the *CYP735A2* and *HYH* promoters *in vivo*. ChIP was performed with seedlings of Col and the NIGT1.1-OX line. Two different regions were amplified by qPCR with immunoprecipitated DNA. Data are means of four biological samples ± s.d. ** p <0.01 by one-tailed t-test compared with corresponding control values obtained with Col seedlings.

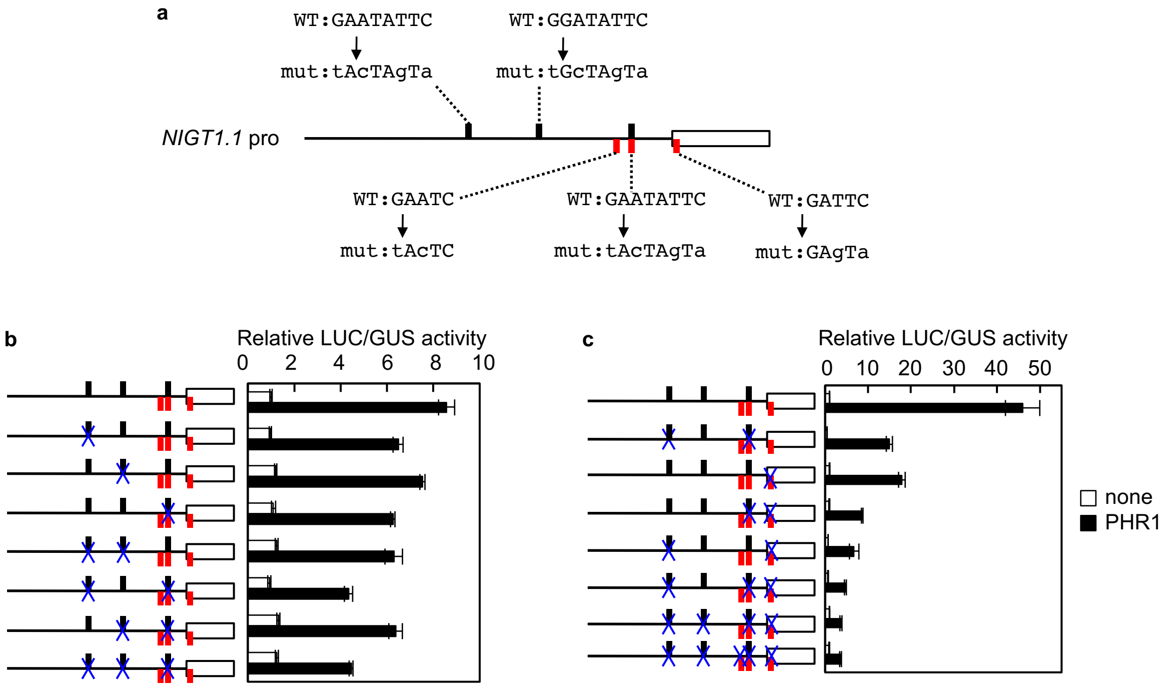

Supplementary Figure 10 | Transactivation of mutant *NIGT1.1* promoters by PHR1. (a) A schematic representation of the 1 kb *NIGT1.1* promoter. A horizontal line and a white box indicate the upstream sequence and the 5' untranslated region, respectively. Black and red boxes indicate the P1BS sequence (5'-GNATATNC-3') and the conserved NIGT1-binding sequences (5'-GAATC-3' and 5'-GAATATTC-3'), respectively. Mutated sequences (mut) are shown below the wild-type (WT) sequences. (**b,c**) Transactivation of mutated *NIGT1.1* promoters by PHR1 in protoplasts. Mutated sites are indicated with blue X. LUC activity was normalised with GUS activity, and data are means ± s.d. of three biological replicates.

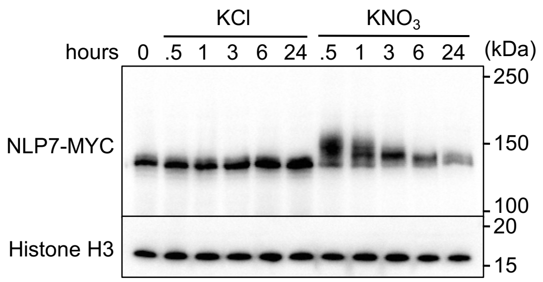

Supplementary Figure 11 | Decreases in the NLP7 protein level after nitrate treatment. Seedlings expressing MYC-tagged NLP7[3] were grown in nitrogen-free 1/10MS solution supplemented with 0.5 mM ammonium succinate [1/10 MS salts, 0.1 g l^{-1} MES-KOH (pH5.7), 0.5% sucrose, 0.5 mM ammonium succinate] for 4 days and treated with 10 mM KCl or KNO_3. Seedlings were collected at indicated time points after the onset of the treatment and used for immunoblot analysis with anti-MYC or anti-Histone H3 antibodies. NLP7 proteins in the active form (retarded bands) more rapidly disappeared, compared with ones in the inactive form.

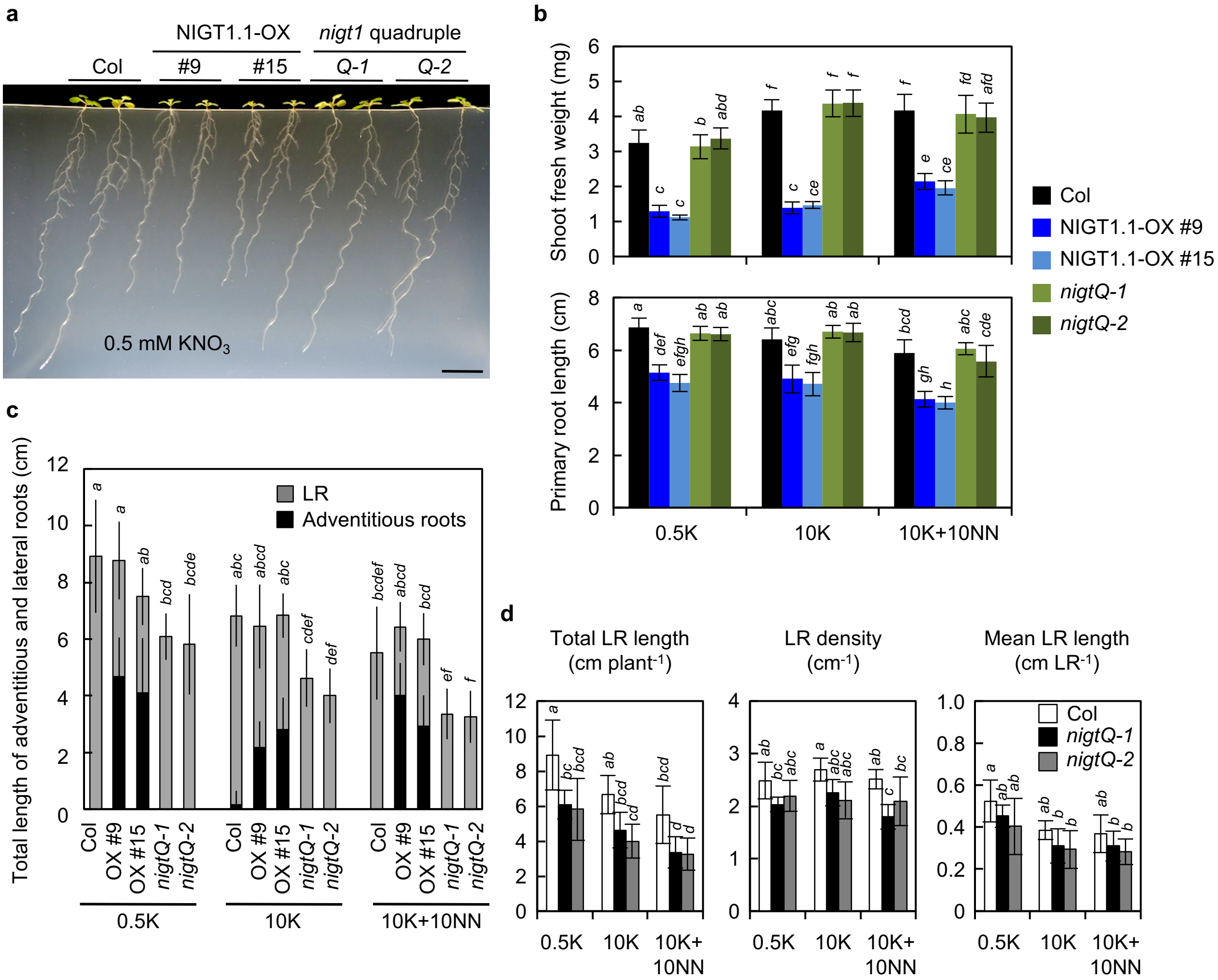

Supplementary Figure 12 | Growth of NIGT1.1-overexpressing plants and *nigt1* quadruple mutants. (**a**) Image of seedlings grown on a plate containing 0.5 mM KNO_3 as N source. Scale bar, 1 cm. Shoot fresh weight and primary root length (**b**), total length of adventitious roots and lateral roots (LR) (**c**), and total LR length, LR density, and mean length of LR (**d**) of Arabidopsis Col, NIGT1.1-OX, and *nigt1* mutants grown under three different N-nutrient conditions (0.5K, 0.5 mM KNO_3; 10K, 10 mM KNO_3; 10K+10NN, 10 mM KNO_3+10 mM NH_4NO_3). Seedlings were initially grown on 1/2MS plates containing 9.4 mM KNO_3 and 10.3 mM NH_4NO_3 as N sources for 3 days, and then grown on test plates containing the indicated N source for 6 days. NIGT1.1-OX plants developed unusually long adventitious roots. Values are means ± s.d. (n=8). Bars not sharing the same letter designation are significantly different from each other (Tukey's HSD, p <0.05). In **c**, the statistical test was performed for the total length of adventitious and lateral roots.

Supplmentary Table 1. Cytokinin contents

pmol g^{-1} FW	Parental line (4xNRE-min-GUS)		NLP6-SUPRD #7		NLP6-SUPRD #14		Col		*nlp6 nlp7-1*		*nigtQ-3*		*nigtQ-3+NIGT1.2*		GFP-OX		NIGT1.2GFP-OX	
	KCl	KNO_3	KCl	KNO_3	KCl	KNO_3	KCl	KNO_3	KCl	KNO_3	KCl	KNO_3	KCl	KNO_3	KCl	KNO_3	KCl	KNO_3
tZ	N.D.	1.080 ± 0.174	N.D.	0.185 ± 0.048	0.028 ± 0.055	0.207 ± 0.054	0.138 ± 0.048	0.917 ± 0.088	0.024 ± 0.049	0.209 ± 0.162	0.027 ± 0.053	0.883 ± 0.104	0.035 ± 0.048	1.034 ± 0.073	0.085 ± 0.058	0.931 ± 0.087	N.D.	0.587 ± 0.099
tZR	0.384 ± 0.054	13.024 ± 1.270	0.563 ± 0.060	2.337 ± 0.330	0.539 ± 0.082	2.950 ± 0.271	0.866 ± 0.131	13.244 ± 0.768	0.280 ± 0.019	4.656 ± 0.208	0.607 ± 0.117	13.797 ± 0.547	0.387 ± 0.055	12.322 ± 0.483	0.274 ± 0.171	9.350 ± 0.234	0.507 ± 0.036	8.371 ± 0.301
tZRPs	2.039 ± 0.295	62.875 ± 5.681	2.447 ± 0.227	11.716 ± 0.732	2.366 ± 0.092	14.422 ± 1.041	2.972 ± 0.454	45.753 ± 3.624	1.473 ± 0.210	19.228 ± 1.267	3.167 ± 0.150	68.848 ± 3.023	1.620 ± 0.146	52.452 ± 2.730	2.055 ± 0.279	56.292 ± 3.314	3.057 ± 0.501	44.819 ± 4.076
cZ	0.394 ± 0.053	0.305 ± 0.045	0.499 ± 0.107	0.247 ± 0.057	0.292 ± 0.041	0.216 ± 0.017	0.684 ± 0.040	0.324 ± 0.047	0.416 ± 0.045	0.152 ± 0.053	0.531 ± 0.068	0.282 ± 0.029	0.599 ± 0.079	0.142 ± 0.135	0.540 ± 0.078	0.250 ± 0.063	0.169 ± 0.195	0.123 ± 0.142
cZR	0.783 ± 0.136	0.586 ± 0.069	0.852 ± 0.056	0.613 ± 0.070	0.884 ± 0.050	0.589 ± 0.047	1.026 ± 0.097	0.762 ± 0.043	0.655 ± 0.107	0.475 ± 0.035	0.961 ± 0.090	0.615 ± 0.069	0.787 ± 0.027	0.566 ± 0.048	0.617 ± 0.162	0.700 ± 0.107	1.036 ± 0.148	0.863 ± 0.214
cZRPs	4.662 ± 0.272	2.423 ± 0.144	5.782 ± 0.569	2.907 ± 0.143	5.248 ± 0.186	2.501 ± 0.115	5.539 ± 0.247	2.555 ± 0.136	3.987 ± 0.202	1.841 ± 0.088	5.797 ± 0.538	2.497 ± 0.066	4.549 ± 0.277	2.045 ± 0.176	4.274 ± 0.190	2.483 ± 0.237	7.415 ± 0.516	3.558 ± 0.083
DZ	N.D.	N.D.	N.D.	N.D.	N.D.	N.D.	N.D.	N.D.	0.072	N.D.	N.D.	N.D.	N.D.	N.D.	N.D.	N.D.	N.D.	N.D.
DZR	N.D.	0.227 ± 0.031	N.D.	N.D.	N.D.	N.D.	N.D.	0.344 ± 0.023	N.D.	N.D.	N.D.	0.255 ± 0.039	N.D.	0.210 ± 0.015	N.D.	0.102 ± 0.027	N.D.	N.D.
DZRPs	N.D.	0.681 ± 0.112	N.D.	0.116 ± 0.015	N.D.	0.158 ± 0.033	0.089 ± 0.015	0.648 ± 0.051	N.D.	0.135 ± 0.007	N.D.	0.730 ± 0.053	N.D.	0.602 ± 0.048	N.D.	0.402 ± 0.020	N.D.	0.325 ± 0.061
iP	0.463 ± 0.554	0.641 ± 0.474	0.450 ± 0.528	0.683 ± 0.461	0.451 ± 0.307	0.728 ± 0.199	0.827 ± 0.097	0.622 ± 0.078	0.385 ± 0.449	0.175 ± 0.350	0.909 ± 1.054	1.642 ± 0.482	0.914 ± 0.072	1.226 ± 0.359	1.551 ± 0.144	1.404 ± 0.557	1.767 ± 0.337	0.990 ± 1.353
iPR	0.222 ± 0.071	0.736 ± 0.036	0.224 ± 0.027	0.403 ± 0.078	0.224 ± 0.065	0.278 ± 0.058	0.455 ± 0.068	1.148 ± 0.141	0.183 ± 0.025	0.574 ± 0.022	0.256 ± 0.028	0.780 ± 0.063	0.277 ± 0.024	0.631 ± 0.106	0.125 ± 0.106	0.697 ± 0.124	0.482 ± 0.135	1.057 ± 0.113
iPRPs	8.260 ± 0.773	29.220 ± 2.078	7.599 ± 0.428	13.323 ± 1.288	6.861 ± 0.197	10.440 ± 1.023	16.620 ± 1.121	36.881 ± 2.360	6.870 ± 0.809	32.559 ± 1.063	11.222 ± 0.749	45.613 ± 2.847	9.233 ± 0.640	32.888 ± 1.573	14.473 ± 0.816	35.240 ± 3.318	15.145 ± 1.691	36.537 ± 2.446
tZ7G	1.178 ± 0.169	4.477 ± 0.249	1.444 ± 0.122	1.737 ± 0.115	1.395 ± 0.042	1.822 ± 0.017	1.834 ± 0.110	4.204 ± 0.190	1.091 ± 0.154	1.943 ± 0.089	1.332 ± 0.097	3.604 ± 0.166	1.372 ± 0.031	5.074 ± 0.183	1.282 ± 0.213	4.322 ± 0.150	0.896 ± 0.080	2.991 ± 0.102
tZ9G	0.246 ± 0.103	1.199 ± 0.248	0.380 ± 0.024	0.407 ± 0.075	0.201 ± 0.067	0.340 ± 0.037	0.448 ± 0.040	0.889 ± 0.190	0.167 ± 0.088	0.555 ± 0.113	0.139 ± 0.161	0.911 ± 0.098	0.316 ± 0.088	1.138 ± 0.153	0.212 ± 0.032	1.082 ± 0.046	0.090 ± 0.064	0.425 ± 0.134
tZOG	1.484 ± 0.159	5.685 ± 0.311	1.598 ± 0.193	1.962 ± 0.366	1.437 ± 0.231	1.803 ± 0.111	2.584 ± 0.292	5.40 ± 0.487	1.439 ± 0.303	3.037 ± 0.382	2.168 ± 0.453	5.600 ± 0.549	1.715 ± 0.333	6.732 ± 0.422	2.301 ± 0.276	9.145 ± 1.366	2.029 ± 0.551	8.351 ± 0.125
cZOG	7.268 ± 0.494	6.382 ± 0.917	9.290 ± 1.201	7.887 ± 0.733	8.233 ± 0.553	6.519 ± 0.355	8.088 ± 0.624	5.768 ± 0.928	6.220 ± 0.856	5.166 ± 0.231	5.975 ± 0.897	3.797 ± 0.561	7.989 ± 0.891	5.725 ± 0.276	7.233 ± 1.215	5.749 ± 0.922	4.818 ± 0.465	5.059 ± 0.963
tZROG	0.019 ± 0.039	0.375 ± 0.077	0.049 ± 0.033	0.082 ± 0.055	0.038 ± 0.028	0.057 ± 0.045	0.122 ± 0.030	0.532 ± 0.037	0.047 ± 0.035	0.112 ± 0.033	0.049 ± 0.034	0.299 ± 0.088	0.044 ± 0.026	0.281 ± 0.088	0.056 ± 0.039	0.336 ± 0.064	0.062 ± 0.074	0.078 ± 0.098
cZROG	0.431 ± 0.048	0.467 ± 0.056	0.415 ± 0.025	0.398 ± 0.049	0.462 ± 0.016	0.413 ± 0.030	0.570 ± 0.046	0.538 ± 0.031	0.349 ± 0.058	0.328 ± 0.021	0.386 ± 0.043	0.381 ± 0.035	0.373 ± 0.041	0.339 ± 0.035	0.348 ± 0.030	0.364 ± 0.068	0.365 ± 0.092	0.388 ± 0.033
tZRPsOG	N.D.	N.D.	N.D.	N.D.	N.D.	N.D.	N.D.	0.071 ± 0.008	N.D.	N.D.	N.D.	N.D.	N.D.	N.D.	N.D.	N.D.	N.D.	N.D.
cZRPsOG	N.D.	N.D.	N.D.	N.D.	N.D.	N.D.	N.D.	N.D.	N.D.	N.D.	N.D.	N.D.	N.D.	N.D.	N.D.	N.D.	N.D.	N.D.
DZ9G	N.D.	N.D.	N.D.	N.D.	N.D.	N.D.	N.D.	N.D.	N.D.	N.D.	N.D.	N.D.	N.D.	N.D.	N.D.	N.D.	N.D.	N.D.
iP7G	106.665 ± 5.996	110.080 ± 2.521	85.748 ± 3.400	82.763 ± 2.025	73.019 ± 4.185	72.792 ± 3.633	94.843 ± 2.322	93.157 ± 2.279	91.659 ± 3.060	96.398 ± 2.955	96.447 ± 2.128	98.204 ± 2.697	86.118 ± 3.911	90.078 ± 3.277	101.625 ± 5.550	113.177 ± 5.441	81.982 ± 5.689	87.289 ± 4.285
iP9G	2.284 ± 0.082	2.398 ± 0.153	1.685 ± 0.089	1.731 ± 0.099	1.432 ± 0.101	1.421 ± 0.115	1.758 ± 0.060	1.925 ± 0.077	2.042 ± 0.113	2.126 ± 0.077	1.844 ± 0.067	1.963 ± 0.150	1.854 ± 0.103	1.828 ± 0.071	2.096 ± 0.049	2.249 ± 0.220	1.352 ± 0.115	1.499 ± 0.099
tZ-type	5.350 ± 0.732	88.715 ± 6.592	6.481 ± 0.327	18.426 ± 1.343	6.003 ± 0.176	21.602 ± 1.377	8.965 ± 0.854	70.943 ± 4.787	4.521 ± 0.466	29.740 ± 1.165	7.488 ± 0.473	93.942 ± 3.119	5.488 ± 0.265	79.034 ± 3.270	6.265 ± 0.571	81.458 ± 3.798	6.641 ± 1.144	65.622 ± 4.057
iP-type	117.893 ± 6.386	143.075 ± 2.455	95.706 ± 3.014	98.903 ± 2.188	81.986 ± 4.350	85.660 ± 3.185	114.503 ± 2.591	133.733 ± 4.550	101.140 ± 3.345	131.833 ± 4.203	110.678 ± 2.402	148.203 ± 5.742	98.395 ± 3.827	126.651 ± 4.872	110.870 ± 5.987	152.768 ± 9.255	100.728 ± 6.902	127.371 ± 6.850
tZ-type precursor	2.423 ± 0.347	75.899 ± 6.701	3.010 ± 0.285	14.053 ± 0.934	2.905 ± 0.100	17.372 ± 1.293	3.838 ± 0.536	58.998 ± 4.304	1.752 ± 0.198	23.884 ± 1.446	3.773 ± 0.063	82.645 ± 3.498	2.006 ± 0.160	64.774 ± 3.094	2.329 ± 0.435	65.642 ± 3.313	3.565 ± 0.503	53.190 ± 4.246
iP-type precursor	8.482 ± 0.763	29.956 ± 2.072	7.823 ± 0.415	13.726 ± 1.361	7.084 ± 0.189	10.718 ± 1.033	17.075 ± 1.137	38.029 ± 2.459	7.054 ± 0.795	33.133 ± 1.060	11.478 ± 0.730	46.393 ± 2.893	9.510 ± 0.651	33.519 ± 1.637	14.598 ± 0.847	35.938 ± 3.425	15.627 ± 1.705	37.594 ± 2.401

4xNRE-ming-GUS, The parental line of NLP6-SUPRD lines. N.D., not detected.
tZ, *trans*-zeatin; tZR, tZ riboside; tZRPs, tZ ribotides; cZ, *cis*-zeatin; cZR, cZ riboside; cZRPs, cZ ribotides; DZ, dihydrozeatin; DZR, DZ riboside; DZRPs, DZ ribotides; iP, N^6-(Δ^2-isopentenyl)adenine; iPR, iP ribosidel iPRPs, iP ribotides; tZ7G, tZ-7-N-glucoside; tZ9G, tZ-9-glucoside; tZOG; tZ-O-glucoside; cZOG, cZ-O-glucoside; tZROG, tZR-O-glucoside; cZROG, cZR-O-glucoside; DZ9G, DZ-9-N-glucoside; iP7G, iP-7-N-glucoside; iP9G, iP-9-N-glucoside.

Supplementary Table 2. Primer list

Amplicon	AGI code	Sequence
**Cloning for transient assay**		
NRT2.1 promoter	At1g08090	CGCAAGCTTCACGTCAGCGAGATTGATCGA<br>CGTCCATGGTTATAAAATATTTCAAGTTTCT
NRT2.1 promoter (NIGT1-site_mut1)	At1g08090	GCAAGATGAGGGAAGGGTAGAGCG<br>TTTTCGAGTACTCCATTTTCGCCGCAAGCATCT<br>GAAAATGGAGTACTCGAAAATTAATATTTAAA<br>CGTCCATGGTTATAAAATATTTCAAGTTTCT
NRT2.1 promoter (NIGT1-site_mut2)	At1g08090	GCAAGATGAGGGAAGGGTAGAGCG<br>TCTTTGGAGAGTATCAACGGTTAACTATCGATT<br>CCGTTGATACTCTCCAAAGAATGCTTTTTTCA<br>CGTCCATGGTTATAAAATATTTCAAGTTTCT
NRT2.1 promoter (NIGT1-site_mut3)	At1g08090	GCAAGATGAGGGAAGGGTAGAGCG<br>TGAGATTTGAGTAAAGTTTATTTGATCGATGTA<br>TAAACTTTACTCAAATCTCAAACTTGCAAAGA<br>CGTCCATGGTTATAAAATATTTCAAGTTTCT
NRT2.1 promoter (NLP-site_D1mut)	At1g08090	CGCAAGCTTCACGTCAGCGAGATTGATCGA<br>ATTAGTTTTATCGTCAAATTGCTTACGATTGCAT<br>AATTTGACGATAAAACTAATGTGCAGCTAAGGC<br>CGTCCATGGTTATAAAATATTTCAAGTTTCT
NRT2.1 promoter (NLP-site_P1mut)	At1g08090	CGCAAGCTTCACGTCAGCGAGATTGATCGA<br>ATCACCACATGGTGCCGATATCCTTCCAAATGAC<br>TATCGGCACCATGTGGTGATAAGCGAGAGACTAGG<br>CGTCCATGGTTATAAAATATTTCAAGTTTCT
NRT2.1 promoter (NLP-site_P2mut)	At1g08090	CGCAAGCTTCACGTCAGCGAGATTGATCGA<br>TTCCTAGTATCGCGCTTATCACCAAAGGTTGC<br>GATAAGCGCGATACTAGGAAGATGCTTGCGG<br>CGTCCATGGTTATAAAATATTTCAAGTTTCT
NRT2.1 promoter (NLP-site_P1P2mut)	At1g08090	CGCAAGCTTCACGTCAGCGAGATTGATCGA<br>ATCGCGCTTATCACCACATGGTGCCGATATCCTTCCAAATGAC<br>CCATGTGGTGATAAGCGCGATACTAGGAAGATGCTTGCGG<br>CGTCCATGGTTATAAAATATTTCAAGTTTCT
NIGT1.1 promoter	At1g25550	CTCGGATCCGAATACATACTCAAAATTAGACACTT<br>GTGCCATGGTGAACAGATCTTAGACTCGTTTTTG
NIGT1.2/HHO2 promoter	At1g68670	CTCGGATCCGAGGAATATGAAAGGTCGGATATG<br>GTGCCATGGTTATTCAAAAGGGACTAAGAACCA
NIGT1.3/HHO1 promoter	At3g25790	CTCGGATCCGGATATAACAACAGCATAGCATAT<br>GTGCCATGGTGGTTTAAGGGACAAAACAAAAAACC
NIGT1.4/HRS1 promoter	At1g13300	CTCGGATCCAATGAAGGTGTTATTAGTAGTGCC<br>GTGCCATGGTGATACTTTAGGGACTTAATTTTAA
NIGT1.1 promoter mut_P1BSn<br>-655 to -648 from ATG<br>GAATATTC to TACTAGTA	At1g25550	CTCGGATCCGAATACATACTCAAAATTAGACACTT<br>GTGGAGTACTAGTACTCGAGAGTAGGGGAGAAACTAAG<br>CTCGAGTACTAGTACTCCACTTTTGCCAAAAACTTCTT<br>GTGCCATGGTGAACAGATCTTAGACTCGTTTTTG
NIGT1.1 promoter mut_P1BS3<br>-502 to -495 from ATG<br>GGATATTC to TGCTAGTA	At1g25550	CTCGGATCCGAATACATACTCAAAATTAGACACTT<br>GAAGAATACTAGCAATAGAGATTGGAATCAAGATATG<br>TCTCTATTGCTAGTATTCTTCACTCGTTTGTTATTACAA<br>GTGCCATGGTGAACAGATCTTAGACTCGTTTTTG
NIGT1.1 promoter mut_P1BSc<br>-299 to -292 from ATG<br>GAATATTC to TACTAGTA	At1g25550	CTCGGATCCGAATACATACTCAAAATTAGACACTT<br>CAGGGATACTAGTAGGGGATATGTGGATTCCTTCCTTT<br>ATCCCCTACTAGTATCCCTGTATCAAACAAACCTCAGC<br>GTGCCATGGTGAACAGATCTTAGACTCGTTTTTG
NIGT1.1 promoter mut_NBS2<br>-315 to -311 from ATG<br>GAATC to TACTC	At1g25550	CTCGGATCCGAATACATACTCAAAATTAGACACTT<br>GATATGTGGAGTACTTCCTTTCCATATTCTATCGAGC<br>AAGGAAGTACTCCACATATCCCCGAATATTCTCCCT<br>GTGCCATGGTGAACAGATCTTAGACTCGTTTTTG
NIGT1.1 promoter mut_NBS1<br>-202 to -198 from ATG<br>GATTC to GAGTA	At1g25550	CTCGGATCCGAATACATACTCAAAATTAGACACTT<br>CTAAAACCTACTCTTCTTGATGTTTAGATTTATTCGG<br>TCAAGAAGAGTAGGTTTTAGGTACGCGACGGCCCGA<br>GTGCCATGGTGAACAGATCTTAGACTCGTTTTTG
NIGT1.1 promoter mut_NBS2 on mut P1BSc	At1g25550	CTCGGATCCGAATACATACTCAAAATTAGACACTT<br>GATATGTGGAGTACTTCCTTTCCATATTCTATCGAGC<br>AAGGAAGTACTCCACATATCCCCTACTAGTATCCCT<br>GTGCCATGGTGAACAGATCTTAGACTCGTTTTTG
NIGT1.1 CDS	At1g25550	TATGGATCCATGGGTATGATGATGTTCAAGAGCGG<br>TCTAGGCCTTGAAAGAGGAAGAAGGTGTGG
NIGT1.2/HHO2 CDS	At1g68670	TATGGATCCATGGGTATGATGGTGGAGATGGATTAC<br>TCTAGGCCTTGACACAGGAGTAGAAGTATTTGT
NIGT1.3/HHO1 CDS	At3g25790	TATGGATCCATGGGTATGATTAAAAAGTTCAGCAATATGG<br>TCTAGGCCTATTATTCTTGACGTAATGATTACGG
NIGT1.4/HRS1 CDS	At1g13300	TATGGATCCATGGGTATGATCAAGAACTTAAGTAATATG<br>TCTAGGCCTTGATATTATTTTTGCATCTTTCGT
LBD37 CDS	At5g67420	ATATCCATGGATGAGCTGCAATGGTTGC<br>GGGCAGGCCTAACAAAAAGGTTAAGCAAC
PHR1 CDS	At4g28610	GTGCCATGGAGGCTCGTCCAGTTCATAGA<br>GTCAGGCCTATTATCGATTTTGGGACGCTTTGGCTG
**RT-qPCR**		
NIGT1.1	At1g25550	TCGATGGTTTAACTAATGACGAAGT<br>TTACTACCGGAGTAGCTGGTCTTC
NIGT1.1 (endogenous only)	At1g25550	ACACACACACCACACCTTCT<br>ACGAACATTCTTGGTTACAAAACA
NIGT1.2/HHO2	At1g68670	TAGACTTCACACAAGAAGGCCAG<br>AATTGTGGTTGTTGCTGATTCC
NIGT1.3/HHO1	At3g25790	GGCAGAAGAGGGCAGAGAAA<br>ATGAGGGCCACCAAGTTGTT

Supplementary Table 2. Primer list (continued)

Amplicon	AGI code	Sequence
**RT-qPCR**		
NIGT1.4/HRS1	At1g13330	CTAACAACGGAAACTCTCAAACG
		CGGTAGTCTTGCCCGTAGAGTA
**RT-qPCR**		
NRT2.1	At1g08090	TGAGCAGGAGAAGCAGAAGA
		TTGTTGGGTGTGTTCTCAGG
NAR2.1/NRT3.1	At5g50200	CTACGCGGTTGATGCCATTG
		GTCGGTGCTCTGTCCATAGG
CYP735A2	At1g67110	TGGACTCTCATGCTCCTTGC
		ACAACTTGCCGGACCTCAT
HYH	At3g17609	CAGAGTATCAGCGCAACAAGC
		TCTCTTCGAGCTGGTCATTGT
UBQ10[a]	At4g05320	GGCCTTGTATAATCCCTGATGAATAAG
		AAAGAGATAACAGGAACGGAAACATAGT
IPS1	At3g09922	AGACTGCAGAAGGCTGATTCAGA
		TTGCCCAATTTCTAGAGGGAGA
PEX4[a]	At5g25760	CTGCGACTCAGGGAATCTTCTAA
		TTGTGCCATTGAATTGAACCC
**RT-PCR**		
NIGT1.1	At1g25550	TGGAACCAATCACCAGATCC
		AGTAGAAGATGTTGCGGGAGAT
NIGT1.2/HHO2	At1g68670	TGATGGTGGAGATGGATTACG
		ACCACCCTTGTTGGCTACATCGGA
NIGT1.3/HHO1	At3g25790	CGAGCTTCCTCTTTGCGTAG
		CTTTTCTTGTGAGCGATCCTG
NIGT1.4/HRS1	At1g13330	GTCGCAATTGCATAGACGCTTC
		GTAGAAGAAGACATCGCCGG
EF1a	At5g60390	ATGCCCCAGGACATCGTGATTTCAT
		TTGGCGGCACCCTTAGCTGGATCA
**EMSA probes**		
NRT2.1 promoter fragment p1_1st	At1g08090	CACGTCAGCGAGATTGATCG
		AGGAAACAGCTATGACCATGCAAAGAAAAACGGCAAACTAAATA
NRT2.1 promoter fragment p1_2nd	At1g08090	CACGTCAGCGAGATTGATCG
		Biotin-ACACAGGAAACAGCTATGACCATG (BIO-M13)
NRT2.1 promoter fragment p2_1st	At1g08090	CTGTTTTATTTAGTTTGCCGTTTTTC
		AGGAAACAGCTATGACCATGTAGTTGTCTCTTCAAATTGCTTAC
NRT2.1 promoter fragment p2_2nd	At1g08090	CTGTTTTATTTAGTTTGCCGTTTTTC
		Biotin-ACACAGGAAACAGCTATGACCATG (BIO-M13)
NRT2.1 promoter fragment p3_1st	At1g08090	GCAATCGTAAGCAATTTGAAGAGA
		AGGAAACAGCTATGACCATGACACTTAATTTGAACTGCTGTAAC
NRT2.1 promoter fragment p3_2nd	At1g08090	GCAATCGTAAGCAATTTGAAGAGA
		Biotin-ACACAGGAAACAGCTATGACCATG (BIO-M13)
NRT2.1 promoter fragment p4_1st	At1g08090	CATGCTGTTACAGCAGTTCAAATT
		AGGAAACAGCTATGACCATGAATGACCGCTCTACCCTTCCCTCA
NRT2.1 promoter fragment p4_2nd	At1g08090	CATGCTGTTACAGCAGTTCAAATT
		Biotin-ACACAGGAAACAGCTATGACCATG (BIO-M13)
NRT2.1 promoter fragment p5_1st	At1g08090	GCAAGATGAGGGAAGGGTAGAGCG
		AGGAAACAGCTATGACCATGTGTTATAAAATATTTCAAGTTTCT
NRT2.1 promoter fragment p5_2nd	At1g08090	GCAAGATGAGGGAAGGGTAGAGCG
		Biotin-ACACAGGAAACAGCTATGACCATG (BIO-M13)
NRT2.1 promoter fragment p5-1_1st	At1g08090	CACGTCAGCGAGATTGATCG
		AGGAAACAGCTATGACCATGCAACGGTTAACTATCGATTTAAATAT
NRT2.1 promoter fragment p5-1_2nd	At1g08090	CACGTCAGCGAGATTGATCG
		Biotin-ACACAGGAAACAGCTATGACCATG (BIO-M13)
NRT2.1 promoter fragment p5-2_1st	At1g08090	AATATTTAAATCGATAGTTAACCGTTG
		AGGAAACAGCTATGACCATGCGATGTCTGAATATTAATCACACG
NRT2.1 promoter fragment p5-2_2nd	At1g08090	AATATTTAAATCGATAGTTAACCGTTG
		Biotin-ACACAGGAAACAGCTATGACCATG (BIO-M13)
NRT2.1 promoter fragment p5-3_1st	At1g08090	ATTGTCAACACTGGCTAGGAACCC
		AGGAAACAGCTATGACCATGTGTTATAAAATATTTCAAGTTTCT
NRT2.1 promoter fragment p5-3_2nd	At1g08090	ATTGTCAACACTGGCTAGGAACCC
		Biotin-ACACAGGAAACAGCTATGACCATG (BIO-M13)
OsNIGT1_1st		CGACGACACCTTCATTTTTATTTTC
		AGGAAACAGCTATGACCATGTTCGTGGGGTGAGGAAGGAAGGAA
OsNIGT1_2nd		CGACGACACCTTCATTTTTATTTTC
		Biotin-ACACAGGAAACAGCTATGACCATG (BIO-M13)
NRT2.1 Proximal_1st	At1g08090	GTCTAGACTGCAGCTGTGCGAGGATATCGGCAACCTTTGG
		GCAAGCTTGTCAGCATGCGGATCTTCCTAGTCTCTCGCTTA
NRT2.1 Proximal_2nd		Biotin-GTCTAGACTGCAGCTGTGCGA (BIO-OligoZ)
		Biotin-GCAAGCTTGTCAGCATGCGGA (BIO-oligoY)
NRT2.1 Distal1_1st	At1g08090	GTCTAGACTGCAGCTGTGCGATCCTATATATGCAATCGTAAG
		GCAAGCTTGTCAGCATGCGGACATTAGTTGTCTCTTCAAATTG
NRT2.1 Distal1_2nd		Biotin-GTCTAGACTGCAGCTGTGCGA (BIO-OligoZ)
		Biotin-GCAAGCTTGTCAGCATGCGGA (BIO-oligoY)
NRT2.1 Distal2_1st	At1g08090	GTCTAGACTGCAGCTGTGCGATTTGAAGAGACAACTAATGTG
		GCAAGCTTGTCAGCATGCGGATTTAGAGTGGCCTTAGCTGC
NRT2.1 Distal2_2nd		Biotin-GTCTAGACTGCAGCTGTGCGA (BIO-OligoZ)
		Biotin-GCAAGCTTGTCAGCATGCGGA (BIO-oligoY)
**Cloning for recombinant expression**		
NIGT1.1 DBD	At1g25550	CGTCCATGGCTGGGGGTGGAAAAGAGTTTGAGGAG
		CGTCTCGAGACCACCGGTCCTTACTACCGGAGT
**Binary plasmid construction**		
NIGT1.1 CDS	At1g25550	CGTCCATGGCTATGTTCAAGAGCGGTGACATGGAT
		TCTAGGCCTTGAAAGAGGAAGAAGGTGTGG

Supplementary Table 2. Primer list (continued)

Amplicon	AGI code	Sequence
**ChIP-qPCR**		
NRT2.1 pro_1	At1g08090	TGACACGTCAGCGAGATTGA<br>CGGTGTGAAAAATTGAAATCGTTGA
NRT2.1 pro_2	At1g08090	GATGAAGACATGTACACACACGA<br>ACATACAGTGATACAGGATAGGCT
NRT2.1 pro_3	At1g08090	TGGTGAAAGAGGACCAAACTTT<br>GCTCACCAGTAGAATCACCCA
NRT2.1 pro_4	At1g08090	GGGTAGAGCGGTCATTTGGA<br>GCTCACCAGTAGAATCACCCA
NAR2.1 pro_1	At5g50200	CAGTGAACGGTTACCTGAGGA<br>AGGGTTGATGTTTGTAGGTCTGT
NAR2.1 pro_2	At5g50200	TGAAAGCAAGACCCTAAACCCA<br>GCCAAAGGATGAGATATGAGTATTTGT
NIGT1.1 pro_1	At1g25550	TGCTGATCCCGATCGTTGT<br>CCATGGATGTGAAGACCACCA
NIGT1.1 pro_2	At1g25550	ACATCATCGCCAGCTCGATA<br>GGGCAGAGAAAGAGGATTGGT
NIGT1.2/HHO2 pro_3	At1g68670	GGAATCGAACCACCAAAGAA<br>GCTAAATGTTCACGCGCATA
NIGT1.2/HHO2 pro_4	At1g68670	ACGTCAGTTCCAGCCCATAC<br>GAAGTGGAAGAGAATCGAGGTC
NIGT1.3/HHO1 pro_5	At3g25790	CTCGAGACTACGAATAGGTGGA<br>TATGTTCGGGTGATGTCCAA
NIGT1.3/HHO1 pro_6	At3g25790	CCCAAATAAATATAAAGTTCACACGA<br>GGCTCTATAGGGTTTCTCAAGG
NIGT1.4/HRS1 pro_7	At1g13300	GGGCGACAGGAAAGAAACT<br>TCCTCATGCATCATTCCAAC
NIGT1.4/HRS1 pro_8	At1g13300	GGTCGTCTAATAAGTATCCA<br>AGGATCAGTATTTTGAGTGA
IPS1 pro_9	At3g09922	CCTGACAGCTCAAGGCATTA<br>TGATGGAAACAAGGGGAGTC
IPS1 pro_10	At3g09922	GTGGTTGCATGCTTGGTTTA<br>TTAGGGTAAAATGGGGCATC
CYP735A2 _c1	At1g67110	GAATGTGGTAATAGCGATAATGTAGACA<br>CTTATCTACTTGGGTGATTGAAATC
CYP735A2 _c2	At1g67110	CTAGATAGCAACCAACTTTCACCAA<br>CAAGATTTTGAGAAAGAGAGAAAGAGAT
HYH _h1	At3g17609	TGTTTATGAGTGATTTCCCGC<br>AGTCAATCATTCCGAAGCATTT
HYH _h2	At3g17609	CATGCAAATAGATACACACACACATA<br>CTCAAAGTCTGCAACTCGTTTCA
**Genotyping of T-DNA lines**		
T-DNA allele of GABI_267G03	At1g25550	CCCATTTGGACGTGAATGTAGACAC (GABI LBP)<br>AGTAGAAGATGTTGCGGGAGAT
WT allele for GABI_267G03	At1g25550	TGGAACCAATCACCAGATCC<br>AGTAGAAGATGTTGCGGGAGAT
T-DNA allele of SALK_044835C	At1g68670	AGCAATCGCAGTCGCATACGCAT<br>ATTTTGCCGATTTCGGAAC-(LBb1.3)
WT allele for SALK_044835C	At1g68670	AGCAATCGCAGTCGCATACGCAT<br>ACCACCCTTGTTGGCTACATCGGA
T-DNA allele of SALK_070096	At1g68670	TGATGGTGGAGATGGATTACG<br>ATTTTGCCGATTTCGGAAC (LBb1.3)
WT allele for SALK_070096	At1g68670	TGATGGTGGAGATGGATTACG<br>GTTTTCTATGCGTATGCGACTG
T-DNA allele of SAIL_28_D03	At3g25790	GAAATGGATAAATAGCCTTGCTTC (pCSA110_LB3)<br>CTTTTCTTGTGAGCGATCCTG
WT allele for SAIL_28_D03	At3g25790	CGAGCTTCCTCTTTGCGTAG<br>CTTTTCTTGTGAGCGATCCTG
T-DNA allele of WiscDsLoxHS231_10C	At3g25790	TGATCCATGTAGATTTCCCGGACATGAAG (L4)<br>CTTTTCTTGTGAGCGATCCTG
WT allele for WiscDsLoxHS231_10C	At3g25790	CGAGCTTCCTCTTTGCGTAG<br>CTTTTCTTGTGAGCGATCCTG
T-DNA allele of SALK_067074	At1g13300	ATTTTGCCGATTTCGGAAC (pROK2_LBb1.3)<br>TCTTCTTTTTCTTCCACTTGCGTAC
WT allele for SALK_067074	At1g13300	GTCGCAATTGCATAGACGCTTC<br>TCTTCTTTTTCTTCCACTTGCGTAC
T-DNA allele (*nlp6*) of SALK_036557	At1g64530	GTGCTGCAGTTATCAATTATCGATTTTGGGACGCTT<br>CTCGGTACCAGGGCAGTTCTTTGCTAATTGGAT
WT allele (*NLP6*) for SALK_036557	At1g64530	GTGAGGCCTAGTTACTGTGCCATCAAAGCCAAG<br>TCCTTAGAGATTTGTGCTGGATGA
T-DNA allele (*nlp7-1*) of SALK_026134	At4g24020	GTGCTGCAGTTATCAATTATCGATTTTGGGACGCTT<br>GTCAGGCCTCAATTCTCCAGTGCTCTCGCAGGA
WT allele (*NLP7*) for SALK_026134	At4g24020	GTTGAATTCGCCACTGTTAACGGTGTGGTTAAG<br>GTCAGGCCTCAATTCTCCAGTGCTCTCGCAGGA
T-DNA allele (*phr1*) of SALK_067629C	At4g28610	GCGTGGACCGCTTGCTGCAACT (pROK2_LBb1)<br>GTGCTGCAGTTATCAATTATCGATTTTGGGACGCTT
WT allele (*PHR1*) for SALK_067629C	At4g28610	GTGCCATGGAGGCTCGTCCAGTTCATAGA<br>GTGCTGCAGTTATCAATTATCGATTTTGGGACGCTT
T-DNA allele (*phl1*) of SAIL_731_B09	At5g29000	GTCGGATCCATGACTCTGGCTAATGATTTCGGA<br>TAGCATCTGAATTTCATAACCAATCTCGATACAC (pCSA110-LB3)
WT allele (*PHL1*) for SAIL_731_B09	At5g29000	GTCGGATCCATGACTCTGGCTAATGATTTCGGA<br>GTCAGGCCTATCTTCTCTGACACGTTTCCTTGA

[a]Czechowski T, Stitt M, Altmann T, Udvardi MK, Scheible WR. Genome-wide identification and testing of superior reference genes for transcript normalization in Arabidopsis. Plant Physiol. 139, 5-17 (2005)

Supplementary References

1. Konishi, M. & Yanagisawa, S. Emergence of a new step towards understanding the molecular mechanisms underlying nitrate-regulated gene expression. *J. Exp. Bot.* **65,** 5589–5600 (2014).
2. Winter, D. *et al.* An "Electronic Fluorescent Pictograph" browser for exploring and analyzing large-scale biological data sets. *PLoS One* **2,** (2007).
3. Liu, K. H. *et al.* Discovery of nitrate-CPK-NLP signalling in central nutrient-growth networks. *Nature* **545,** 311–316 (2017).
