# Supplementary material for: A NIGT1-centred transcriptional cascade regulates nitrate signalling and incorporates phosphorus starvation signals in Arabidopsis
Source: Nat Commun. 2018 Apr 10;9:1376. doi: 10.1038/s41467-018-03832-6 (PMC5893545; doi:10.1038/s41467-018-03832-6)
Supplement: Supplementary file 2 — Description of Additional Supplementary Files [file 41467_2018_3832_MOESM2_ESM.pdf]

**Description of Additional Supplementary Files**

Supplementary Data 1.

List of nitrate-inducible genes whose expression is repressed by NIGT1.2 overexpression.

Supplementary Data 2.

List of nitrate-repressed genes whose expression is reduced by NIGT1.2 overexpression.
